# A decision-theoretic framework for wastewater treatment performance assessment based on a fuzzy parameterized fuzzy hypersoft set approach

**DOI:** 10.1038/s41598-025-07896-5

**Published:** 2025-07-01

**Authors:** Muhammad Haris Saeed, Fatima Razaq, Muhammad Saeed, Muhammad Salman Habib, Seung-June Hwang

**Affiliations:** 1 Department of Chemistry, University of Management and Technology, 54700 Lahore, Punjab Pakistan; 2 Department of Mathematics, University of Management and Technology, 54700 Lahore, Punjab Pakistan; 3 Institute of Knowledge Services, Center for Creative Convergence Education, Hanyang University ERICA Campus, 15588 Ansan, Gyeonggi-do South Korea; 4 College of Business and Economics, Hanyang University ERICA Campus, 15588 Ansan, Gyeonggi-do South Korea

**Keywords:** Wastewater, Fuzzy Set, Sustainability, Optimization, Decision Making, Hypersoft Set, Environmental Policy Design, Environmental impact, Applied mathematics, Computational science, Sustainability

## Abstract

Abstract The economic development of a country is profoundly influenced by how it protects and utilizes its natural resources, one of which is wastewater, with emphasis on environmental sustainability. For this multi-factorial sustainability analysis, this study introduces a novel Fuzzy Parameterized Fuzzy Hypersoft Set (FPFHSS) structure to develop a comprehensive decision-making and performance evaluation system for wastewater treatment facilities. The practicality of the designed system is explored by highlighting its ability to evaluate urban projects and work as a decision-making system for environmental policy design. The study introduces an algorithm based on the hypersoft structure, allowing the division of attributes into sub-attributes for a more concise analysis. The sub-parametric values are first parameterized into fuzzy numbers and then evaluated based on their relative importance. With proper fuzzy parameterization of each sub-attribute, novel specialized FPFHSS based Technique of Ordered Preference Similar to Ideal Solution (TOPSIS) and Multi-Objective Optimization on the basis of a Ratio Analysis (MULTIMOORA) approaches are developed that provide a versatile analysis in addition to the newly developed algorithm. With these 3 specialized systems, 4 case studies were developed based on 19 pseudo-realistic environmental, social, technical, and economic factors each simulating a different scenario faced by nations highlighting sustainability, technical performance and the environment allowing for informed decision-making while addressing uncertainty making them highly suitable as a computational AI solution for real-data analysis. This fuzzy analysis offers a reference for making informed decisions in the context of environmental remediation and complex scenario simulations.

## Introduction

Natural resource conservation and sustainable use, combined with environmental sustainability, are major factors in a nation’s economic prosperity. Scientific and technological developments have made it possible to use these natural resources more effectively all over the world. Wastewater is one of these priceless resources, and it’s crucial to a nation’s environmental and ecological sustainability^1^. The efficient utilization of these resources has become more imminent than ever due to the exponential increase in population around the world with the rapid depletion of natural resources. For this purpose, a rational decision support system for decision-making is necessary to address the numerous factors while calculating the risk of each factor to tackle the possible water shortage crises. The system is needed to evaluate the effectiveness of currently in use wastewater treatment facilities while taking into account the socioeconomic aspects associated with these operations.

When discussing wastewater resource management, selecting the best technology is eminent while ensuring budgeting, social, economic, and technical factors while acknowledging the public benefit to be obtained from the project. To this end, policy makers are the decision makers directly responsible for selecting the technology to be used for the wastewater treatment project, as they are considered experts in terms of technical knowledge, expertise, and competence in the relevant field^2^. During this decision-making process, a number of uncertainties arise as the factors considered for the wastewater treatment project are not always in the same format (i.e., linguistic variables, discrete numbers, interval-based variables). These uncertainties can’t be overlooked as they may cause errors in forecasting analysis during the performance analysis while considering the factors associated with the environmental, social, and risk-associated factors^3^.

To address all these concerns, the motivation of the presented work is to develop a performance-analytic decision support system that can address the uncertainty developed during the optimal technology selection or evaluation process for the wastewater treatment facility. Because it consists of qualities, alternatives, dimensions, and goals that need to be met, this multi-criteria decision-making problem might be seen as fuzzy for this reason^4^. The objective of decision makers is to quantify alternatives based on their qualitative and quantitative nature to access the optimal results in a particular setting. Linguistic values are scaled to mathematical syntax to convert the qualitative values of an attribute to a crisp value that can be analyzed^5^. This allows for a proper dominant/recessive ranking of the factors that are being considered for the final decision-making problem. Also, these attributes that express the alternative are not equally responsible for a particular quality of the system to be quantified. It is essential to introduce some weight terms in this regard as each factor does not have equal importance, while the experts or decision-makers judging the technology may not have equivalent expertise in a particular phase of the decision-making process. Linguistic terms allow room for uncertainty to develop, so it is addressed in the form of Fuzzy Sets whose mathematical operations are clearly defined and have been used in literature many times^6^. Fuzzy sets allow the evaluation of criteria while incorporating the weights defined for decision making while presenting uncertainty concisely^7^. It also has the ability to deal with incomplete data and the uncertainty associated with them, as will be required when analyzing linguistic terms obtained from an expert’s conscious judgment.

Fuzzy set-based systems have been used in literature for various environmental analytic applications. For the assessment of water quality, Ocampo-Duque et al.^8^ provided an approach based on fuzzy inference systems. Gharibi et al.^9^ presented a new fuzzy logic-based water quality index, which is a complete artificial intelligence process for environmental index creation. This innovative method is intended for the routine assessment of surface water quality. A two-tuple fuzzy linguistic Delphi technique, introduced by Montes et al.^10^, expands on this by allowing judges with varying degrees of competence to evaluate surveys through decision-making. For switched chaotic systems, Vadivel et al.^11^ presents a sampled-data controller based on T-S fuzzy logic that ensures exponential stability. It uses a novel Lyapunov-Krasovskii method that uses LMIs to formulate conditions and includes switching signal data. The effectiveness of the controller is confirmed in several chaotic systems. In a study by Yang et al.^12^, an approach to multi-objective fuzzy linear fractional programming is presented and used to solve an optimization problem about planting structures in agriculture. Li and Yang^13^ explored the idea of fuzzy probabilistic covering-based rough sets for three-way decisions and examine its use in credit assessment. Using grey fuzzy reasoning grade analysis as the primary methodology for optimization, Tran et al.^14^ examined the study, which focuses on multi-objective optimization of the drilling process for carbon fiber-reinforced polymer materials. Karamoozian et al.^15^ suggests a new way to identify safety risks in construction that may be uncertain. It makes use of the IVIF-DEMATEL, Choquet integral and IF-TOPSIS methods from fuzzy logic to list and choose the primary safety factors prioritized by experts. The use of a case study demonstrates that the approach improves the reliability of assessing risks. Karamoozian et al.^16^ outlines a unique blend of fuzzy techniques for judging possible risks in renewable energy (RE) investment projects. The risk factors are analyzed and ranked by using interval type-2 hesitant fuzzy DEMATEL-based ANP (DANP) and the QUALIFLEX approach for all four main dimensions. It proves that the model is steady and the results supply valuable information for those involved in finance and policy.

In order to determine the weighting of the evaluation criteria in fuzzy synthetic evaluation as part of the assessment of water quality, (Zou et al.^17^ investigates the application of the entropy approach. Using fuzzy logic and Complex Event Processing (CEP) technology, the FUME decision support system for urban areas addresses air quality concerns^18^. In a study by Das et al.^19^, an adaptive controller using fuzzy logic facilitates the integration of a photovoltaic system with a three-phase shunt hybrid active filter, therefore improving industrial power quality. In order to improve biogas production in wastewater treatment, Waewsak et al.^20^ uses a neural-fuzzy control system to monitor process reaction and govern an anaerobic hybrid reactor. Karamoozian and Wu^21^ introduces a hybrid fuzzy technique to rank risks in construction industry supply chains affected by the COVID-19 pandemic. Integrating interval-valued intuitionistic fuzzy DEMATEL, Choquet integral and IF-TOPSIS, the model highlights and ranks the main risk areas, including shortages of supplies and limits on funding due to COVID-19 restrictions. Karamoozian et al.^22^ introduces a hybrid DEMATEL-ANP and LCA strategy to decide on the best type of pipe for hydrocarbon pipeline projects. Technical, economic and environmental measures are reviewed, confirming that GRE uses the least energy. During the years 2001 to 2006, Chunping et al.^23^ evaluated Beijing’s municipal solid waste transfer stations using fuzzy synthetic evaluation techniques and environmental monitoring. A hybrid method based on an adaptable neuro-fuzzy inference system is presented by Abbas et al.^24^ to efficiently handle load disaggregation for residential consumers. Fuzzy logic is a crucial part of the control system in the design and verification of a LQR controller for big wind turbines, as discussed in Jeon and Paek^25^. Saeed et al.^26^ proposed a new approach is presented utilizing fuzzy hypersoft sets to support renewable energy management in Turkey. The approach addresses uncertainty and the effects of expert bias using sub-parametric analysis and entropy techniques to support clearer evaluation and development of renewable systems. Despite relying on data and being subjective, it could be a good solution for numerous cases and even more applications with hybrid upgrades. Using cross-scale injection moulding, Shan et al.^27^ presents a unique method for multi-objective optimization in the production of protein electrophoresis microfluidic chips using fuzzy rule-based techniques. Fuzzy hypersoft structures have been applied in many different contexts, much as the work on fuzzy sets in environmental rehabilitation. A sophisticated fuzzy hypersoft set-based algorithm was used by Saeed et al.^28^ to assess the effectiveness of solid waste management. Comparably, similar structures have been documented in the literature as supporting systems for diagnostics for a variety of viral and infectious illnesses^29–31^.

As discussed above, wastewater treatment presents itself as an intricate multiattribute problem with complex parameters of both linguistic and discrete nature^32^. In the situation where there are some experts, a challenge presents itself, as contradictory opinions about a particular aspect of the project are pretty common as each person’s judgmental consciousness is based on different ways of thinking while analyzing a particular attribute^33^. The literature review revealed a significant amount of work that allows a combined analysis of social, environmental, and economic factors to be made to determine the optimal system for wastewater treatment at a particular location. In order to solve a multi-criterion decision making problem based on an expert’s opinion for the analysis of the factors mentioned above, some of these approaches use analytical network theory (ANP) and analytical hierarchy process (AHP) while taking into account a small number of factors to determine the environmental and economic impacts of a wastewater treatment facility^34–36^. This type of analysis involves using crisp values, which isn’t possible in most real-world applications as data available to analyze the parameters is heterogenous. The studies mentioned above provide a direction that can be used to analyze data but don’t address various data types when encountering a real-life problem, particularly when considering the optimal selection of technology for a wastewater facility under a dynamic and uncertain environment.

The structure of the paper is organized into several key sections to ensure a comprehensive presentation of the proposed research. Section 1 provides the introduction, which is further divided into five subsections: Subsection 1.1 presents a detailed literature review, Subsection 1.2 outlines the identified research gap, Subsection 1.3 highlights the novelty of the proposed methodology, Subsection 1.4 discusses the motivation behind the study, and Subsection 1.5 summarizes the main contributions of the paper. Section 2 describes the problem statement along with the dataset utilized in this study. Section 3 introduces the concept of the FPFHSS, which forms the theoretical foundation of the research. Section 4 focuses on the design of tailored decision-making algorithms within FPFHSS environments. Section 5 presents the simulation of these algorithms for the optimal selection of wastewater treatment facilities across various scenarios. Section 6 includes the results of the study, offering a comparative analysis, detailed discussions, and final experimental outputs. Finally, Section 7 concludes the paper with a summary of the key findings and a discussion of the study’s contributions.

### Literature review

Various scenarios have been subjected to multi-criteria decision-making procedures, which are determined by the complexity and type of data available. The ways in which each technique manipulates the data to reach a conclusion vary. Because of this, each of the techniques developed has its own set of advantages and disadvantages. One of the techniques called TODIM (”Interactive and multicriteria decision-making” translated from the Portuguese language) was used for the psychological analysis of the behavior of individuals in a risky and uncertain environment^37^. In the literature, linguistic data was transformed to create a business evaluation model for an energy company using a hybrid TODIM approach based on the intuitionistic fuzzy set^38^. In order to manage decision-making difficulties in unpredictable contexts and choose the best storage solutions for carbon capture, a different hybrid TODIM approach was created based on a fuzzy set^39^.

Although there has been numerous research on the Analytic Hierarchy Process (AHP), there are still some misconceptions about it that need to be cleared up. Among these myths are things like rank reversals, the limitations of a 9-point rating system, and the functions of redundancy, intransitivities, and inconsistencies. It has also been investigated how to incorporate MCDM approaches into practical decisions^40^^41^. has created integrated decision-making procedures for certain applications, such the assessment of Six Sigma initiatives. With each MCDM strategy having pros and cons, the difficult part of the process is figuring out which one is best for a particular problem^42^. One normalization strategy that has been utilized to improve the effectiveness of MCDM systems is the Performance by Similarity to Ideal Solution (TOPSIS) methodology^43^, the idea that we tried to improve on in our study. The linguistic intersection method (LIM) is one of the new decision-making approaches that have been created to solve the challenges of selecting the optimal MCDM methodology for a specific case^44^. Integrated MCDM techniques based on type-2 fuzzy sets have been developed for use in energy option selection^45^. These methods aim to include ambiguity and personal preferences into the decision-making process. Among the MCDM techniques that have proven useful for supplier selection procedures in sectors like the petroleum industry are fuzzy and intuitionistic fuzzy TOPSIS with flexible entropy weighting^46^. Within the framework of AHP approaches, fuzzy-based strategies have been employed to address uncertainty in risk assessment processes^47^. Novel techniques have been developed for process parameter optimization in applications like Electrical Discharge Machining (EDM), such as merging ENTROPY-COPRAS methodologies with ENTROPY-TOPSIS methodologies^48^. Using MCDM approaches, green supplier selection has also been studied, with an emphasis on environmental factors^49^. The interval type-2 fuzzy prioritized Choquet normalized weighted BM operators have been developed for this purpose since we believe that environmental capabilities should be considered in supplier selection processes.

As discussed above, Wang and Elhag^50^ introduced the fuzzy TOPSIS approach, which is based on alpha level sets and has shown to be more successful than earlier iterations of fuzzy TOPSIS. Jahanshahloo et al.^51^ extended TOPSIS for decision-making with interval data and presented an algorithm for determining the most preferred option among all options. The interval-valued fuzzy sets-based TOPSIS approach for decision analysis was elaborated upon by Chen and Tsao^52^. Ballı and Korukoğlu^53^ used the TOPSIS technique in conjunction with decision makers’ subjective evaluations to select an operating system for corporate computer systems. An extended TOPSIS technique was presented by Ye^54^ to address partner selection problems when there is insufficient and unclear information. In group decision making, this approach uses interval-valued intuitionistic fuzzy numbers. According to crucial success indicators, the major air carriers’ ranking was ascertained by Torlak et al.^55^ through the use of the fuzzy TOPSIS approach for business rivalry analysis in the Turkish domestic airline market. Using both the TOPSIS and fuzzy TOPSIS techniques, Rathod and Kanzaria^56^ examined PCM selection in a multiple attribute decision-making setting. By Chi and Liu^57^, the TOPSIS method was extended to Interval Neutrosophic Sets to handle multiple attribute decision-making problems where the attribute weights are unknown. Chen^58^ developed an inclusion-based TOPSIS technique with interval-valued intuitionistic fuzzy sets for group decision-making involving many criteria. Kutlu Gündoğdu and Kahraman^59^ introduced the Spherical Fuzzy TOPSIS Method, which expands the application of TOPSIS to spherical fuzzy sets. These studies demonstrate the TOPSIS approach’s adaptability and flexibility to various decision-making scenarios. For our study, we tried to develop a hybrid TOPSIS approach that utilizes the FPFHSS data structure.

Similarly, the MULTIMOORA technique is a versatile instrument that has been extended and adjusted in various ways to address a range of decision-making settings. Brauers and Zavadskas^60^ introduced the ratio system and Reference Point Theory as an alternative technique within the MULTIMOORA framework. Baležentis and Zeng (2013)^61^ modified the method to include type-2 fuzzy sets in order to account for uncertain evaluations while generating multi-criteria decisions. Fuzzy set theory and the MULTIMOORA technique were used by Liu et al.^62^ to create a novel risk prioritization model for evaluating the risk of failure modes. Gou et al.^63^ introduced the double hierarchy hesitant fuzzy linguistic term set to enhance the decision-making process, in contrast to already-in-use methods such as hesitant fuzzy linguistic TOPSIS. Zhao et al.^64^ combined continuous weighted entropy and interval-valued intuitionistic fuzzy sets with the MULTIMOORA method for Failure Mode and Effect Analysis (FMEA). Steviç et al.^65^ added approximations for supplier selection in a construction company to the COPRAS and MULTIMOORA techniques. A study by Fattahi and Khalilzadeh^66^ revealed a fuzzy MULTIMOORA method for risk assessment was introduced. Fuzzy weighted risk priority values based on cost, profit, and time considerations are taken into consideration. For multi-expert decision-making tasks, (Wu et al.^67^ introduced the Probabilistic Linguistic MULTIMOORA technique, which incorporates subjective judgments and correlation coefficients between criteria. Huang et al.^68^ developed the Pythagorean Fuzzy MULTIMOORA approach, which makes use of new distance measurements and a score function, to handle multicriteria decision-making under Pythagorean fuzzy set information. Based on an improved MULTIMOORA approach, Lin et al.^69^ suggested an image fuzzy MCDM model for vehicle sharing station site selection. These studies demonstrate the MULTIMOORA method’s adaptability and efficacy in a variety of decision-making contexts, as well as its robustness and use in a variety of circumstances.

The weight criteria are among the most significant elements affecting the entire Multi-Criteria Decision Making (MCDM) process. Various methods have been proposed to determine the weight coefficients of the criteria in MCDM. One approach is to assign a weight to each criterion based on its relative importance, ensuring that the sum of the weights is equal to one^70^. Determining criteria weights is a common challenge in MCDM techniques, and numerous approaches have been put forth to address it^71–73^. Using an appropriate method for deciding criteria weights is essential for producing dependable MCDM judgments. A hybrid MCDM technique based on combination weight has been developed^74^ to address several unclear and contradictory criteria in the context of facility layout selection. This reveals the intricacy of MCDM issues and the demand for creative solutions to manage a wide range of criteria. Furthermore, for data-driven decision-making, a multi-criteria approach has been used, in which alternatives are evaluated and ranked according to the weight of each criterion^75^. This illustrates how adaptable MCDM techniques are to many decision-making situations. Additionally, the ranking of alternatives in MCDM processes can be greatly impacted by the criteria weight method selection. For instance, the chosen technique for criterion weight and the particular MCDM method applied affected the vendors’ rankings on a platform for vendor evaluation for the purchase of medical equipment^76^. This emphasizes how important it is to carefully choose and apply criterion weight mechanisms to ensure the validity and reliability of MCDM conclusions.

### Research gap

Despite significant advancements in MCDM techniques such as AHP, TOPSIS, and MULTIMOORA several critical limitations persist. Existing studies have primarily focused on theoretical enhancements and minor methodological improvements, often relying on traditional fuzzy or intuitionistic fuzzy sets. Although extensions using type-2 fuzzy sets, neutrosophic sets, and other soft computing models have been proposed, these methods still struggle to effectively model highly uncertain, hierarchical, and parameterized decision-making scenarios, especially in sustainability focused domains like smart wastewater treatment. In particular, FPFHSS framework offers a promising mechanism for managing multidimensional uncertainty by decomposing attributes into sub-attributes. Despite its potential, its application in practical decision-making has been minimally explored, with existing research primarily focused on basic aggregation operations. Comprehensive studies that integrate FPFHSS with hybrid MCDM techniques such as TOPSIS and MULTIMOORA and employ entropy-based weight determination and simulation analysis are still lacking in the current literature. This study addresses these gaps by introducing a novel FPFHSS-based MCDM framework that integrates TOPSIS and MULTIMOORA with entropy-based weighting and weight simulation. The proposed methodology offers a robust and adaptive tool for tackling complex, real-world decision-making problems, particularly in the selection of sustainable and efficient smart wastewater treatment facilities.

### Novelty of the proposed methodology

Achieving Efficient and sustainable waste management requires navigating a complex landscape of environmental, social, technical, and economic factors, demanding innovative decision-making approaches to improve clarity and effectiveness. While existing literature mainly addresses the theoretical definition of FPFHSS with limited application to aggregation operators, this study pioneers the use of FPFHSS integrated with MCDM techniques specifically TOPSIS and MULTIMOORA augmented by the entropy method for objective weight determination simulation-based analysis.

The FPFHSS approach uniquely decomposes decision attributes into sub-attributes and converts data into fuzzy variables, effectively managing uncertainties inherent in waste management decisions. This precision and adaptability are demonstrated through four practical scenarios:

- **Case# 1** In MCDM, the entropy technique is used to calculate weights because it offers an unbiased, data-driven evaluation of criteria relevance, especially in situations where explicit information is not accessible. There are various reasons why the entropy technique is chosen:
  1. It is grounded in information theory and statistics, ensuring objectivity and minimizing bias.
  2. Normalizing entropy values ensures that the total of the weights is equal to 1, which is essential for MCDM techniques.
  3. It lessens the cognitive strain on decision-makers by streamlining the process of giving weights to multiple variables.
  4. It is advantageous when there are no definite subjective preferences since it derives weights from the data at hand rather than depending on the judgment of experts.
- **Case# 2** In waste management decision-making, stakeholders often regard all factors as equally important. This balanced perspective ensures comprehensive decisions by equally considering social, technical, environmental, and economic aspects. Social factors pertain to community well-being, technical factors to practical capabilities, environmental aspects to sustainability, and economic factors to cost-efficiency. The FPFHSS method is adept at balancing these aspects, ensuring that no factor is overlooked and all are impartially considered.
- **Case# 3** Environmental sustainability is a critical priority in waste management, with agencies focusing on minimizing the ecological impact of waste disposal. The FPFHSS approach supports this by assigning higher weights to environmental factors, ensuring that plant selection aligns with environmental protection objectives.
- **Case# 4** In certain cases, technical and economic factors are prioritized to achieve maximum sustainability and long-term utility. The FPFHSS approach facilitates this by allowing higher weights for these factors, ensuring the selected waste management plant is both efficient and cost-effective over the long term.

**Fig. 1:**
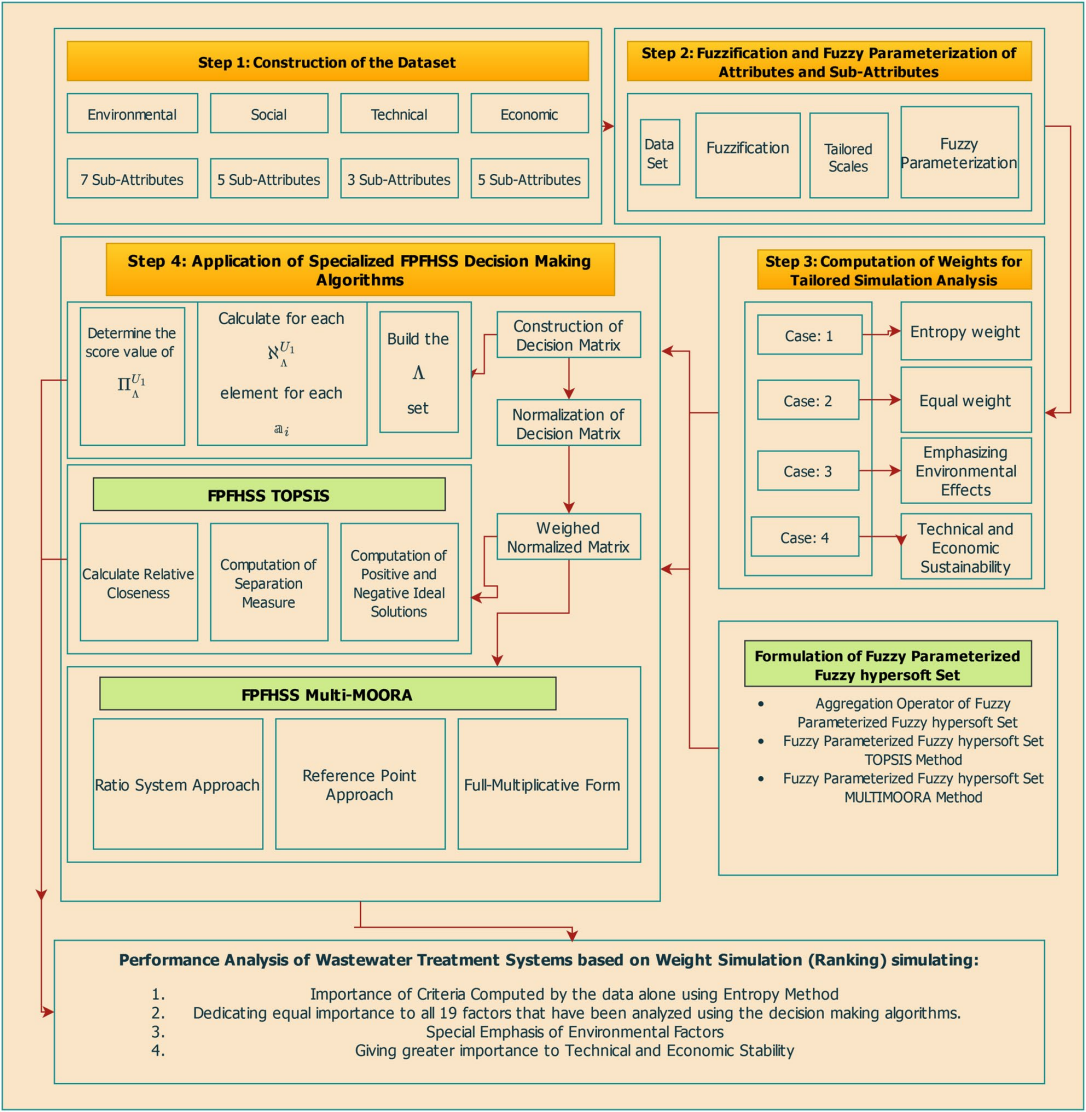
Design of the Study.

Through comprehensive simulation analysis on smart wastewater treatment facilities, the proposed methodology demonstrates its practical effectiveness and flexibility. By bridging the gap between theoretical FPFHSS constructs and real-world MCDM challenges, this study contributes a robust, scalable, and novel framework for sustainable infrastructure planning and environmental decision-making. The study is mapped out in the following manner (Figure 1):

### Motivation

In complex decision-making environments such as those involving environmental sustainability and infrastructure planning the relative importance of evaluation criteria often varies depending on context, stakeholder values, and reference points. These attributes are reference-dependent, meaning that their perceived dominance or relevance changes dynamically across different scenarios. Consequently, assigning appropriate weights to these criteria is critical, as it directly influences the ranking of alternatives and the overall decision outcome. Several limitations arise in such contexts, particularly when formulating policies and selecting sustainable solutions in environmental domains:

1. There is a clear need for an assessment system based on multi-dimensional criteria.
2. Dealing with data in different heterogeneous formats, i.e., linguistic, discrete, or in the form of intervals.
3. A dynamic system needs to be able to deal with uncertain conditions with respect to the environment and human intuition in decision making.
4. Inability to deal with incomplete or insufficient data in the decision-making process.

Motivated by these challenges, this study introduces an advanced decision-making framework that integrates the FPFHSS with hybrid MCDM techniques specifically, TOPSIS and MULTIMOORA augmented by entropy based weighting and simulation analysis. The proposed model is designed to navigate uncertainty, support flexible attribute decomposition, and enable more reliable decisions in sustainability focused applications such as smart wastewater treatment facility selection.

### Main contribution

The primary focus of this article is to develop a fuzzy set theory based system to determine the performance of any environmental remediation project (i.e., wastewater treatment facility etc.) based on fuzzy parameterization of each alternative allowing for better handling of uncertainty as each alternative is expressed within an interval from 0 to 1. The framework is superior to the ones in literature as it allows for a decision-making process with a better rationale and risk aversion factor under an uncertain and complex environment. Considering these limitations, a fuzzy parameterized fuzzy hypersoft set is introduced to compute and handle the multi-dimensional and heterogeneous criteria. The fuzzy parameterization allows for the criteria that are to be considered for the decision-making to be in a homogeneous format and be expressed in the form of fuzzy membership. The criteria are further sub-divided into sub-attributes for a more detailed analysis under diverse environmental conditions provided during the study. The methodology proposed in the manuscript aims to handle factors related to sustainability while providing a versatile system that can serve as an efficient decision support system and a performance evaluation system for environmental remediation projects and policies. The model aims to incorporates how social, political, environmental, and economic factors influence the planning and developmental stage of the wastewater treatment project during the decision-making process. The key contributions of this study are summarized as follows:

- This work pioneers the integration of FPFHSS with two prominent MCDM techniques TOPSIS and MULTIMOORA which have not previously been explored in this context.
- A novel FPFHSS structure is developed that decomposes decision attributes into sub-attributes and maps them into fuzzy intervals, enabling fine-grained representation of complex, real-world decision factors.
- The methodology employs the entropy technique to assign data-driven weights to criteria, reducing subjectivity and enhancing decision reliability when expert judgment is unavailable or uncertain.
- The proposed system effectively handles both qualitative and quantitative data including linguistic, crisp, and interval-valued information making it suitable for real-world smart wastewater treatment decisions.
- The proposed methodology is validated through four decision-making scenarios, each reflecting different stakeholder preferences (e.g., balanced, environmental, economic), to demonstrate the flexibility and adaptability of the framework.
- By applying the model to smart wastewater treatment plant selection, the study bridges theoretical development with practical application, offering a scalable decision-support solution for sustainable infrastructure planning.

## Problem statement

To validate our methodology, let’s consider a bustling metropolitan city, home to a significant population of over 10 million residents, spread across an area of 6 square kilometers. These specifications closely mirror the characteristics of densely populated cities found throughout Europe. In recent years, the population has surged due to substantial in-migration, effectively doubling the city’s headcount. While planned urban centers and developed nations have devised strategies to manage such demographic shifts, it’s a different story in developing countries, where these rapid population surges pose significant challenges. Istanbul serves as a prime example of a city grappling with these issues. In particular, the city has historically experienced abundant water resources, but the situation has taken a turn for the worse due to the sudden influx of residents^77^. As a result, water scarcity has emerged as a pressing concern, prompting government officials to explore wastewater reuse as a viable source of clean water, particularly for non-potable purposes. This predicament is not unique to Istanbul, as similar scenarios are playing out in cities worldwide, particularly in regions of Asia, Africa, and South America. Given these factors, it becomes imperative to establish a system for validating wastewater treatment technologies that can cope effectively with the management of large volumes of wastewater and allow the selection of the most suitable treatment methods for sustainability and efficiency. In particular, the decision-making process should extend its scope to encompass often overlooked factors, such as greenhouse gas emissions, sludge treatment techniques, and the integration of renewable energy sources.

This problem is important because there is a great need for decision aids that are able to address tough, multi-level issues where things can change rapidly. Traditional approaches for making decisions which use simple fuzzy logic, struggle to handle hierarchical structures and changing significance of attributes, particularly for sustainability in infrastructure planning. The key innovation of this study is the creation and use of a new decision-making framework based on FPFHSS, together with the MCDM techniques known as TOPSIS and MULTIMOORA and made more powerful using entropy methods and simulations. With this approach, attributes can be divided into smaller sub-attributes, uncertainties can be fully modeled and ratings can be adapted, making the tool more suitable for reviewing smart wastewater treatment systems.

Within this framework, we evaluate four distinct wastewater treatment systems, each capable of handling volumes exceeding 100,000 cubic meters per day. These alternatives encompass a variety of technologies and methodologies for wastewater treatment. The alternatives include:

1. A2O with pre-clarification (A2O-W/-P) (**A1**)
2. 5-stage Bardenpho with pre- clarification (BP-5-W/-P) (**A2**)
3. A2O without pre-clarification (A2O-W/O-P) (**A3**)
4. Conventional Activated Sludge System with pre-clarification and digester (CAS-W/-P) (**A4**)

**Fig. 2:**
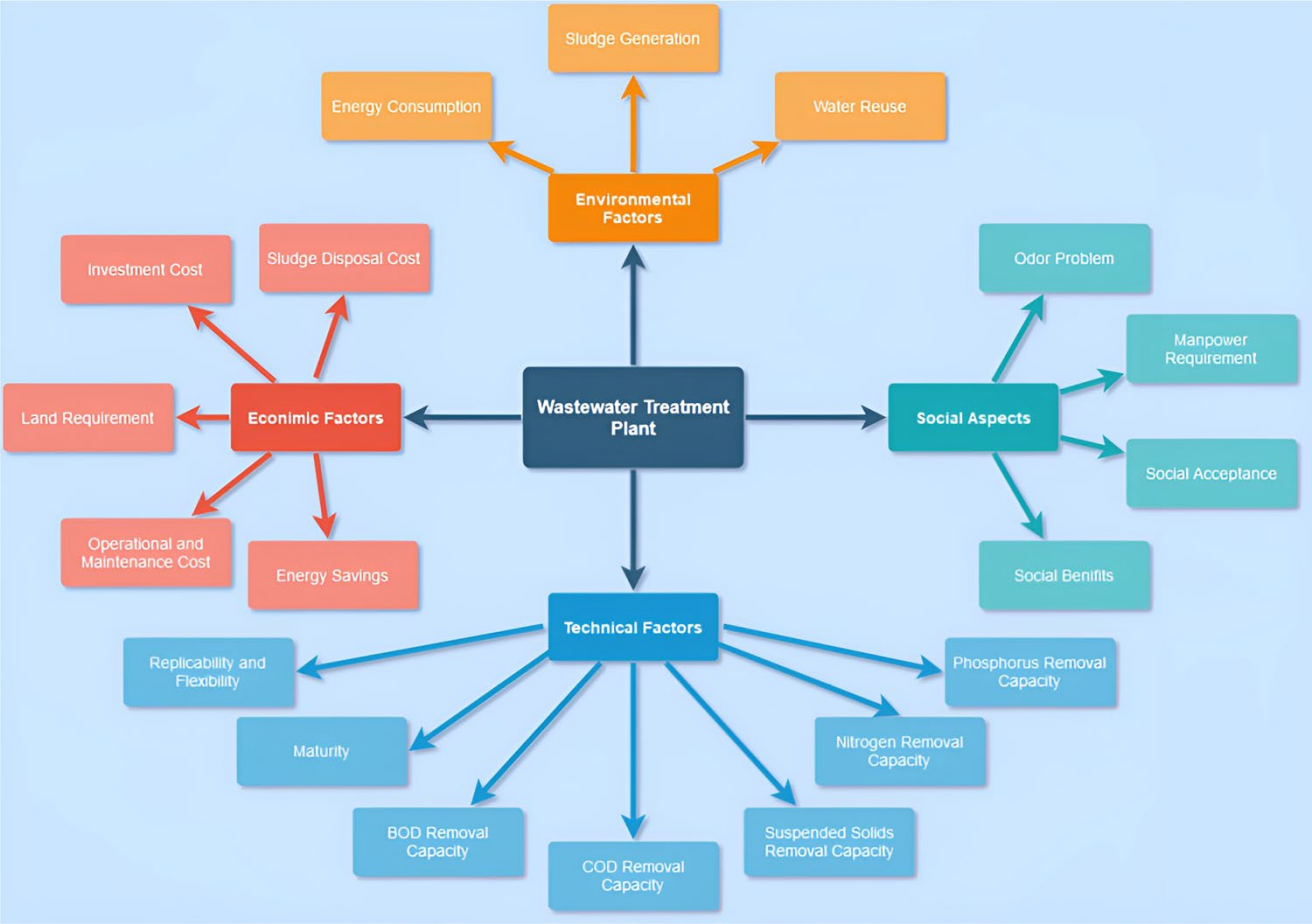
A sub-attributive division of criteria considered for performance analysis.

Now, selecting the Optimal Wastewater Treatment System requires a number of factors to be considered, including technical, social, economic, and environmental factors. The process of treating wastewater is considered costly, complex, and a high-energy demanding process making the factors considered for the decision-making model to be highly strategic as its long-lasting sustainability is in question. A system that addresses 4 major factors with 19 sub-criterion was considered for the analysis that incorporates environmental, social, technical, and economic factors represented in the Figure 2 below:

Considering environmental factors in the study is crucial as the wastewater treatment facility needs to be eco-friendly and doesn’t harm the environment. With this notion in mind, the environmental impacts of the facility were determined using the reusability and conservation of resources. The factors that were considered for the study are:

- Consumption of Energy obtained from the Grid
- The amount of sludge produced during the treatment process
- The degree of reusability of water post-treatment

The Economics of a project is the root cause of evaluating whether a project is a success or failure. For this purpose, the factors considered here include expenditure from the planning and installation phase to the operational phase. The operational phase is where no external investment is required for the operation of the plant. While other factors considered include the cost of land, the cost of sludge disposal, the amount of energy saved and the expenditure for the continuous maintenance of the wastewater treatment facility^78^. The technical performance of the wastewater treatment facility was measured using the degree of efficiency to remove the biological oxygen demand (BOD), chemical oxygen demand (COD), suspended solids (SS), nitrogen (N), and phosphorus (P). The social impact was determined using 4 different parameters: cultural acceptance, the components of the human resource of sustainable development, awareness of the pros and cons of the project, and the responsibilities associated with it. Also, based on the nature of the criteria, they are categorized as a MIN or MAX attribute. A detailed overview of the attributes is presented in Table 1.

**Table 1:** A detailed overview of the attributes selected for performance evaluation process.

**Criteria**	**Labels**	**Attributes**	**Units of Measurement**	**Nature of Attribute**	**Description**
Environmental Factors	A1	Energy Consumption	MW/Day	Min	Energy consumed during a day of operations
	A2	Sludge Generation	Tonn/Day	Min	Sludge generated post processing
	A3	Water Reuse	Litres/Day	Max	Reusable water generate per day
Social Factors	A4	Odor Problem	Reference Scale	Min	Odor from the processing of wastewater
	A5	Manpower Requirement	Job Creation	Max/Min	No. of trained professionals required on-site
	A6	Social Acceptance	Scaled	Max	People’s perception of the plant
	A7	Social Benefits	Scaled	Max	People’s perception of benefits of the plant
Technical Factors	A8	Replicability and Flexibility	Scaled	Max	Load handling and expansion capacity
	A9	Maturity	Scaled	Max	Reliability
	A10	BOD Removal	%age	Max	BOD removal from waste water
	A11	COD Removal	%age	Max	COD removal from waste water
	A12	Suspended Solids Removal	%age	Max	Suspended Solids removal from waste water
	A13	Nitrogen Removal	mg/L	Max	Nitrogen Removal
	A14	Phosphorus Removal	mg/L	Max	Phosphorus Removal
Economic Factors	A15	Energy Savings	MW/Year	Max	Energy savings from waste water
	A16	Operational and Maintainance cost	$$/Year	Min	Operational and maintainance cost of daily operations
	A17	Land Req	sq m	Min	Land req for wastewater treatment facility
	A18	Investmet Cost	Mil $$	Min	Initial Investment Required for installing the plant
	A19	Sludge Disposal cost	$$/Year	Min	Sludge disposal cost

## Fuzzy Parameterized Fuzzy Hypersoft Set (FPFHSS)

Zadeh^79^ developed fuzzy set theory, which extends ordinary set theory to handle uncertainty by allowing partial memberships between 0 and 1, which represent progressive boundaries and ambiguity in the real world. Soft sets were first introduced by Molodsov^80^ in 1999, where they were described as a parameterized family of subsets inside a universal set. A pair $(\mathbb {X}, \mathbb {A})$ is referred to as a soft set in soft set theory. In this case, $\mathbb {A}$ is a set of pairs $(\mathfrak {x}, \mathbb {\alpha })$, which represent elements $\mathfrak {x}$ from $\mathbb {X}$ and their corresponding degrees of membership $\mathbb {\alpha }$ in the interval [0, 1]. By adding sub-attribute functions, Smarandache expanded the idea of soft sets in 2018 to hypersoft sets. Fuzzy parameterized fuzzy hypersoft sets, thus, are sophisticated mathematical structures that incorporate several levels of uncertainty, personalization, and sophisticated information processing. The procedure is divided into the following sections to help with understanding. The word ”fuzzy” firstly suggests that this set functions under the fuzzy logic framework, in which information that is imprecise or unclear can be represented by elements with degrees of membership ranging from 0 to 1. By implying that the membership functions that define this set can be modified using parameters, the term ”parameterized” offers an extra degree of flexibility. We can customize the membership functions to fit particular applications or scenarios thanks to the freedom provided by parameters. Finally, hypersoft introduces the concept of hypersoft computing, which is a sophisticated mathematical framework that expands on soft computing and fuzzy logic. Hypersoft computing is skilled at managing data with high degrees of complexity, contradiction, and ambiguity in addition to imprecision.

Here, some fundamental definitions are provided that are used for the development of a FPFHSS algorithm are presented:

### Definition 3.1

(Fuzzy Set) A fuzzy set $\varphi _\mathfrak {F}$ defined as $\varphi _\mathfrak {F} = \{( R_{\varphi _\mathfrak {F}}(\omega )/\omega : \omega \in \mathfrak {A} \}$, such that $R_{\varphi _\mathfrak {F}}: \mathfrak {A} \rightarrow \mathfrak {I}$ where $R_{\varphi _\mathfrak {F}}$ denotes the belonging value of $\omega \in \varphi _\mathfrak {F}$.

### Definition 3.2

(Fuzzy Parameterized Fuzzy Soft Set) Assume $\Psi _{\varpi }$ is a fuzzy parameterized fuzzy soft set for a set $\mathfrak {P}$ within a set of all parameters $\mathfrak {E}$. Each element $\mathfrak {p}$ in $\mathfrak {P}$ is characterized by two aspects: its membership degree in a fuzzy set ${G_\varpi }(\mathfrak {p}) \in \mathfrak {F}(u)$ and its importance within another fuzzy set ${k_\varpi }(\mathfrak {p}) \in \mathfrak {F}(u)$. The collection comprises pairs indicating the ratio of ${G_\varpi }(\mathfrak {p})$ to $\mathfrak {p}$ and ${k_\varpi }(\mathfrak {p})$.- A fuzzy parameterized fuzzy soft set $\Psi _{\varpi }$ declared as:

$$\begin{aligned} {\Psi _\varpi } = \left\{ {\left( {\frac{{{G_\varpi }(\mathfrak {p})}}{\mathfrak {p}},{k_\varpi }(\mathfrak {p})} \right) ;{G_\varpi }(\mathfrak {p}) \in \mathfrak {F}(u),{k_\varpi }(\mathfrak {p}) \in \mathfrak {F}(u)\; and\; \mathfrak {p} \in \mathfrak {P}} \right\} \\ where \mathfrak {P} \subseteq \mathfrak {E}, {G_\varpi } : \mathfrak {P} \rightarrow \mathfrak {I} and {k_\varpi }: \mathfrak {P} \rightarrow \mathfrak {F}(u). \end{aligned}$$

### Definition 3.3

(Fuzzy Parameterized Fuzzy Hypersoft Set) Let ${\mathfrak {M}_i}, i = 1, 2,..., n$ be parameter-valued sets for parameters ${\hbar _i} \in \mathbb {E}$ (a collection of parameters), where ${\mathfrak {M}_i} \cap {\mathfrak {M}_j} = \emptyset ,{\hbar _i} \ne {\hbar _j}, i \ne j\; and \; \mathfrak {M} = \prod \limits _{i = 1}^n {{\mathfrak {M}_i} = {\mathfrak {M}_1}*{\mathfrak {M}_2}*...*{\mathfrak {M}_n}.}$

A fuzzy parameterized fuzzy hypersoft set $\Pi _{\Lambda }$ described as:

$$\begin{aligned} \Pi _{\Lambda }= \left\{ {\left( {\frac{{\hat{\gamma }}}{{{\zeta _\Lambda }(\hat{\gamma })}},{\chi _\Lambda }(\hat{\gamma })} \right) ;{\zeta _\Lambda }(\hat{\gamma }) \in \mathfrak {F}(u),{\chi _\Lambda }(\hat{\gamma }) \in \mathfrak {F}(u)\; and \; \hat{\gamma }\in \mathfrak {M}} \right\} ,\\ where {\zeta _\Lambda }:\mathfrak {M} \rightarrow \mathfrak {I} with {{\zeta _\Lambda }(\hat{\gamma })} \end{aligned}$$

Since each fuzzy membership relates to $\hat{\gamma }\in \mathfrak {M}$ and ${\chi _\Lambda }:\mathfrak {M} \rightarrow \mathfrak {F}(u)$ is an approximation function with several arguments with ${\chi _\Lambda }(\hat{\gamma }) = \left\{ {\frac{{{\mathfrak {y}_j}}}{{{\sigma _\Lambda }({\mathfrak {y}_j})}},{\mathfrak {y}_j} \in u} \right\}$ as an approximation element of fuzzy hypersoft $\Pi _{\Lambda }$.

### Definition 3.4

(Aggregation of $\Lambda -set$) Let $\Lambda -set$ $\Pi _{\Lambda } \in \Gamma _{\Lambda }$; in that case, a set comprising fuzzy decisions $\Pi _{\Lambda }$ is represented by the symbol $\Pi _{_\Lambda }^{{U_1}} = \left\{ {\aleph _{_\Lambda }^{{U_1}}(\hat{\beta })/\hat{\beta }:\hat{\beta }\in \acute{B}} \right\}$, where $\aleph _{_\Lambda }^{{U_1}}:\acute{B} \rightarrow \mathfrak {I}$ and;

$$\begin{aligned} \aleph _{_\Lambda }^{{U_1}}(\hat{\beta }) = \frac{1}{{|\mathfrak {M}|}}\sum \limits _{\hat{\gamma }\in \mathfrak {M}} {{\zeta _\Lambda }(\hat{\gamma }){\Xi _{{\chi _\Lambda }(\hat{\gamma })}}} (\hat{\beta }) \times \breve{w}_{n}.\\ \end{aligned}$$

For each $\mathfrak {M}$, $|\mathfrak {M}|$ denotes its cardinality.

$$\begin{aligned} {\Xi _{{\chi _\Lambda }(\hat{\gamma })}}(\hat{\beta }) = \left\{ {\begin{array}{*{20}{c}} {{\chi _\Lambda }(\hat{\gamma })\;\; ; \;\; \hat{\beta }\in {\Xi _{{\chi _\Lambda }}}(\hat{\gamma })} \\ {0 \;\; ; \;\; \hat{\beta }\notin {\Xi _{{\chi _\Lambda }}}(\hat{\gamma })} \end{array}} \right. \end{aligned}$$

To calculate the value of $\aleph _{_\Lambda }^{{U_1}}(\hat{\beta })$, the following steps should be followed:

- Begin by selecting parametric tuples that include $\hat{\beta }$ in their approximations. In other words, ensure that the value of ${\Xi _{{\chi _\Lambda }(\hat{\gamma })}}(\hat{\beta })$ matches their corresponding fuzzy grades ${{\chi _\Lambda }(\hat{\gamma })}$.
- Proceed and compute the product of ${{\zeta _\Lambda }(\hat{\gamma })}$ and the outcome of ${\Xi _{{\chi _\Lambda }(\hat{\gamma })}}(\hat{\beta })$. Finally, summarize these items. Take the weight vector and multiply the total of the values. $\breve{W}=[\breve{w}_{1}+\breve{w}_{2}+\breve{w}_{3}+...+\breve{w}_{n}]$, where $\breve{w}_{1}+\breve{w}_{2}+\breve{w}_{3}+...+\breve{w}_{n}=1$.
- Finally, split the calculated total by the cardinality $|\mathfrak {M}|$ of the $\mathfrak {M}$ set.

## Design of tailored decision-making algorithms in fuzzy parameterized fuzzy hypersoft set environments

With the development of the novel FPFHSS mentioned in the previous section, the next section proposes how the designed tailored operation are used to design a custom decision-support system that will later on be used as a performance evaluation system for wastewater treatment facilities.

### Novel decision-making algorithm based on specialized aggregation of $\Lambda -set$ in a fuzzy parameterized fuzzy hypersoft set environment

The fundamental ideas of the $\Lambda -set$ as they were described by Rahman et al.^81^ as a generalisation of the ideas presented in^82–84^ with some changes.

All of the collected $\Lambda -sets$ is signified by $\Gamma _{\Lambda }$.

The fuzzy set, soft set, fuzzy soft set, hypersoft set, fuzzy hypersoft set, and fuzzy parameterized fuzzy soft set are among the subsets that fall under the umbrella of the $\Lambda -set$ notion. Here are some particular $\Lambda -set$ variations:

- The fuzzy parameterized fuzzy soft set is obtained by replacing the hypersoft set with the soft set.
- It takes on the characteristics of a parameterized fuzzy soft set when fuzzy parameterization is removed.
- It becomes a fuzzy soft set by ignoring fuzzy parameterization and replacing the hypersoft setting with the soft option.
- It becomes a soft set if fuzzy parameterization is disregarded, the soft environment takes the place of the hypersoft environment, and fuzzy grades are removed.
- It becomes a fuzzy set if fuzzy parameterization is removed, the hypersoft setting is replaced with the soft setting, and fuzzy approximations are not included.

With the conditions provided, the algorithmic design for decision making using Fuzzy Parameterized Fuzzy Hypersoft Set is explained below:

- **Step 1:** Let’s consider an initial universe represented by $\acute{B}$, where $\acute{B}$ is a set containing elements $\left\{ \mathbb {a}_{1}, \mathbb {a}_{2}, \mathbb {a}_{3}, \ldots , \mathbb {a}_{n}\right\}$. Additionally, a collection of attributes denoted as $\mathbb {E}$, which consists of elements $\left\{ \mathfrak {f}_{1}, \mathfrak {f}_{2}, \mathfrak {f}_{3}, \ldots , \mathfrak {f}_{n}\right\}$.
- **Step 2:** Organize the elements within $\mathbb {E}$ into distinct, non-overlapping sub-classes based on their sub parametric values:
  $$\begin{aligned} \mathbb {E}_{1}= & \left\{ \mathfrak {f}_{11}, \mathfrak {f}_{12}, \mathfrak {f}_{13}, \ldots , \mathfrak {f}_{1n}\right\} \\ \mathbb {E}_{2}= & \left\{ \mathfrak {f}_{21}, \mathfrak {f}_{22}, \mathfrak {f}_{23}, \ldots , \mathfrak {f}_{2n}\right\} \\ \mathbb {E}_{3}= & \left\{ \mathfrak {f}_{31}, \mathfrak {f}_{32}, \mathfrak {f}_{33}, \ldots , \mathfrak {f}_{3n}\right\} \end{aligned}$$
  And so on, until:
  $$\begin{aligned} \mathbb {E}_{n}=\left\{ \mathfrak {f}_{n1}, \mathfrak {f}_{n2}, \mathfrak {f}_{n3}, \ldots , \mathfrak {f}_{nn}\right\} . \end{aligned}$$
- **Step 3:** Build the $\Lambda -set$ $\Pi _{\Lambda }$ using the principles outlined in definition (3.3), and then present it in a tabular format.
- **Step 4:** Using the recommendations in definition (3.4), calculate ${\aleph _{_\Lambda }^{{U_{1}}}}$ for each element $\mathbb {a}_{i}$, where $i = 1, 2,..., k$, from the collection $\acute{B}$.
- **Step 5:** Determine the score value of $\Pi _{_\Lambda }^{{U_{1}}}$ in accordance with the guidelines outlined in Definition (3.4).
- **Step 6:** Choose the option that performs the best under the specified conditions from the set of ${\aleph _{_\Lambda }^{{U_{1}}}}(\mathbb {a}_{i})$, with the highest score value.

### Fuzzy parameterized fuzzy hypersoft set TOPSIS method

According to the referenced source, the TOPSIS approach takes into account two key sets: ${{{\hat{\gamma }}}} = {{{\hat{\gamma }}_1}}, {{{\hat{\gamma }}_2}},..., {{{\hat{\gamma }}_\mathfrak {m}}}$ representing a set of criteria and $\Pi _{\Lambda } = \Pi _{\Lambda _{1}}, \Pi _{\Lambda _{2}},...,\Pi _{\Lambda _\mathfrak {m}}$ representing a set of alternatives. The following is a summary of the TOPSIS method’s procedure:

- **Step 1:** Construct the decision matrix.
  Let $\Pi _{\Lambda } = \{\Pi _{\Lambda _\mathfrak {ij} }\}$ be a decision matrix, where $\mathfrak {i},\mathfrak {j} = 1, 2, ..., n$ for decision maker or expert, then
  1 $$\begin{aligned} \Pi _{\Lambda _{\mathfrak {ij}}} = \left[ {\begin{array}{*{20}{c}} {{{\left( {\frac{{{{\hat{\gamma }}_\mathfrak {i}}}}{{{\zeta _\Lambda }({{\hat{\gamma }}_\mathfrak {i}})}},{\chi _\Lambda }({{\hat{\gamma }}_\mathfrak {i}})} \right) }^{11}}}& {{{\left( {\frac{{{{\hat{\gamma }}_\mathfrak {i}}}}{{{\zeta _\Lambda }({{\hat{\gamma }}_\mathfrak {i}})}},{\chi _\Lambda }({{\hat{\gamma }}_\mathfrak {i}})} \right) }^{12}}}& \cdots & {{{\left( {\frac{{{{\hat{\gamma }}_\mathfrak {i}}}}{{{\zeta _\Lambda }({{\hat{\gamma }}_\mathfrak {i}})}},{\chi _\Lambda }({{\hat{\gamma }}_\mathfrak {i}})} \right) }^{1n}}}\\ {{{\left( {\frac{{{{\hat{\gamma }}_\mathfrak {i}}}}{{{\zeta _\Lambda }({{\hat{\gamma }}_\mathfrak {i}})}},{\chi _\Lambda }({{\hat{\gamma }}_\mathfrak {i}})} \right) }^{21}}}& {{{\left( {\frac{{{{\hat{\gamma }}_\mathfrak {i}}}}{{{\zeta _\Lambda }({{\hat{\gamma }}_\mathfrak {i}})}},{\chi _\Lambda }({{\hat{\gamma }}_\mathfrak {i}})} \right) }^{22}}}& \cdots & {{{\left( {\frac{{{{\hat{\gamma }}_\mathfrak {i}}}}{{{\zeta _\Lambda }({{\hat{\gamma }}_\mathfrak {i}})}},{\chi _\Lambda }({{\hat{\gamma }}_\mathfrak {i}})} \right) }^{2n}}}\\ \vdots & \vdots & \vdots & \vdots \\ {{{\left( {\frac{{{{\hat{\gamma }}_\mathfrak {i}}}}{{{\zeta _\Lambda }({{\hat{\gamma }}_\mathfrak {i}})}},{\chi _\Lambda }({{\hat{\gamma }}_\mathfrak {i}})} \right) }^{n1}}}& {{{\left( {\frac{{{{\hat{\gamma }}_\mathfrak {i}}}}{{{\zeta _\Lambda }({{\hat{\gamma }}_\mathfrak {i}})}},{\chi _\Lambda }({{\hat{\gamma }}_\mathfrak {i}})} \right) }^{n2}}}& \cdots & {{{\left( {\frac{{{{\hat{\gamma }}_\mathfrak {i}}}}{{{\zeta _\Lambda }({{\hat{\gamma }}_\mathfrak {i}})}},{\chi _\Lambda }({{\hat{\gamma }}_\mathfrak {i}})} \right) }^{nn}}} \end{array}} \right] \end{aligned}$$
  and $\mathfrak {W}=[\mathfrak {w}_{1}+\mathfrak {w}_{2}+\mathfrak {w}_{3}+...+\mathfrak {w}_\mathfrak {n}]$ weight vector, where $\Pi _{\Lambda _\mathfrak {ij} }$ and $\mathfrak {w}_{1}+\mathfrak {w}_{2}+\mathfrak {w}_{3}+...+\mathfrak {w}_\mathfrak {n}=1$.
- **Step 2:** In order to facilitate comparisons across various criteria, this phase entails converting diverse attribute dimensions into non-dimensional properties. Since criteria frequently have measurements in different units, the scores in the evaluation matrix $\Pi _{\Lambda }$ must be normalized onto a scale. You can apply a few of the normalizing techniques that were previously discussed. Assume for the moment that we apply the normalizing formula shown below: Let,
  2 $$\begin{aligned} {\mathfrak {R}} =\left[ {\begin{array}{*{20}{c}} {{\mathfrak {r}_{11}}}& {{\mathfrak {r}_{12}}}& {\cdots }& {{\mathfrak {r}_{1\mathfrak {n}}}} \\ {{\mathfrak {r}_{21}}}& {{\mathfrak {r}_{22}}}& {\cdots }& {{\mathfrak {r}_{2\mathfrak {n}}}} \\ {\vdots }& {\vdots }& {\vdots }& {\vdots } \\ {{\mathfrak {r}_{\mathfrak {m}1}}}& {{\mathfrak {r}_{\mathfrak {m}2}}}& {\vdots }& {{\mathfrak {r}_{\mathfrak {mn}}}} \end{array}} \right] = {\left( {\mathfrak {r}_\mathfrak {ij}} \right) _{\mathfrak {m} \times \mathfrak {n}}} \end{aligned}$$
  where, $$\mathfrak {r}_\mathfrak {ij} ={{{\left( {\frac{{{{\hat{\gamma }}_\mathfrak {i}}}}{{{\zeta _\Lambda }({{\hat{\gamma }}_\mathfrak {i}})}},{\chi _\Lambda }({{\hat{\gamma }}_\mathfrak {i}})} \right) }}}$$ $$\mathfrak {i}=1,2,...,\mathfrak {m}; \mathfrak {j}=1,2,...,\mathfrak {n}$$
  3 $$\begin{aligned} \mathfrak {r}_\mathfrak {ij} = \frac{{{\Pi _{\Lambda }}}}{{\sqrt{\sum \limits _{\mathfrak {j} = 1}^\mathfrak {n} {{{(\Pi _{\Lambda })}^2}} } }}, \end{aligned}$$
- **Step 3:** Determine the decision matrix that is weighted and normalized. The following formula yields the weighted normalized value $\mathfrak {v}_\mathfrak {ij}$:
  4 $$\begin{aligned} \mathfrak {v}_\mathfrak {ij} = {\mathfrak {w}_\mathfrak {j}}*\mathfrak {r}_\mathfrak {ij} \quad for \; \mathfrak {i} = 1,..., \mathfrak {m}; \, \mathfrak {j} = 1,...,\mathfrak {n}. \end{aligned}$$
  where $\sum \limits _{\mathfrak {j} = 1}^\mathfrak {n} {{\mathfrak {w}_\mathfrak {j}}} = 1$, is the weight of the $\mathfrak {j}^{th}$ criteria.
- **Step 4:** Obtain the fuzzy positive- and negative-ideal solutions based on intuition. Think of the benefit criterion as $\mathfrak {J}_{1}$ and the cost criterion as $\mathfrak {J}_{2}$. Give the intuitionistic fuzzy positive-ideal solution $\Pi _{\Lambda }^*$ and the intuitionistic fuzzy negative-ideal solution $\Pi _{\Lambda } ^-$. The following is the computation of $\Pi _{\Lambda }^*$ and $\Pi _{\Lambda } ^-$:
  The positive-ideal solution is $\Pi _{\Lambda }^*$, and the negative-ideal solution is $\Pi _{\Lambda }^-$. Next, we derive $\Pi _{\Lambda }^*$ and $\Pi _{\Lambda } ^-$ as follows:
  5 $$\begin{aligned} \Pi _\Lambda ^* = \left( {\frac{{{{\hat{\gamma }}_\mathfrak {i}}}}{{\zeta _\Lambda ^*({{\hat{\gamma }}_\mathfrak {i}})}},\chi _\Lambda ^*({{\hat{\gamma }}_\mathfrak {i}})} \right) ,\Pi _\Lambda ^ - = \left( {\frac{{{{\hat{\gamma }}_\mathfrak {i}}}}{{\zeta _\Lambda ^ - ({{\hat{\gamma }}_\mathfrak {i}})}},\chi _\Lambda ^ - ({{\hat{\gamma }}_\mathfrak {i}})} \right) . \end{aligned}$$
  where,
  6 $$\begin{aligned} & \left( {\frac{{{{\hat{\gamma }}_\mathfrak {i}}}}{{\zeta _\Lambda ^*({{\hat{\gamma }}_\mathfrak {i}})}},\chi _\Lambda ^*({{\hat{\gamma }}_\mathfrak {i}})} \right) = \left( {\mathop {\max }\limits _\mathfrak {i} \left( {\frac{{{{\hat{\gamma }}_\mathfrak {i}}}}{{{\zeta _\Lambda }({{\hat{\gamma }}_\mathfrak {i}})}},{\chi _\Lambda }({{\hat{\gamma }}_\mathfrak {i}})} \right) |\mathfrak {j} \in {\mathfrak {J}_1}} \right) ,\left( {\mathop {\min }\limits _\mathfrak {i} \left( {\frac{{{{\hat{\gamma }}_\mathfrak {i}}}}{{{\zeta _\Lambda }({{\hat{\gamma }}_i})}},{\chi _\Lambda }({{\hat{\gamma }}_\mathfrak {i}})} \right) |\mathfrak {j} \in {\mathfrak {J}_2}} \right) , \end{aligned}$$
  7 $$\begin{aligned} & \quad \left( {\frac{{{{\hat{\gamma }}_\mathfrak {i}}}}{{\zeta _\Lambda ^-({{\hat{\gamma }}_\mathfrak {i}})}},\chi _\Lambda ^-({{\hat{\gamma }}_\mathfrak {i}})} \right) = \left( {\mathop {\min }\limits _\mathfrak {i} \left( {\frac{{{{\hat{\gamma }}_\mathfrak {i}}}}{{{\zeta _\Lambda }({{\hat{\gamma }}_i})}},{\chi _\Lambda }({{\hat{\gamma }}_\mathfrak {i}})} \right) |\mathfrak {j} \in {\mathfrak {J}_1}} \right) ,\left( {\mathop {\max }\limits _\mathfrak {i} \left( {\frac{{{{\hat{\gamma }}_\mathfrak {i}}}}{{{\zeta _\Lambda }({{\hat{\gamma }}_\mathfrak {i}})}},{\chi _\Lambda }({{\hat{\gamma }}_\mathfrak {i}})} \right) |\mathfrak {j} \in {\mathfrak {J}_2}} \right) . \end{aligned}$$
- **Step 5:** Determine the measures for separating the positive and negative ideal solutions. For each alternative, the fuzzy parametrized fuzzy hypersoft set positive-ideal and negative-ideal solutions are found using the separation measures ${\mathfrak {S}_{{\mathfrak {i}^*}}}$ and ${\mathfrak {S}_{{\mathfrak {i}^-}}}$. We utilize the normalized Euclidean distance in this situation.
  8 $$\begin{aligned} {\mathfrak {S}^*}= & {\left( {\frac{1}{{2\mathfrak {n}}}\sum \limits _{\mathfrak {j} = 1}^\mathfrak {n} {{{\left( {\left( {\frac{{{{\hat{\gamma }}_\mathfrak {i}}}}{{{\zeta _\Lambda }({{\hat{\gamma }}_\mathfrak {i}})}},{\chi _\Lambda }({{\hat{\gamma }}_\mathfrak {i}})} \right) - \left( {\frac{{{{\hat{\gamma }}_\mathfrak {i}}}}{{\zeta _\Lambda ^*({{\hat{\gamma }}_\mathfrak {i}})}},\chi _\Lambda ^*({{\hat{\gamma }}_\mathfrak {i}})} \right) } \right) }^2}} } \right) ^{\frac{1}{2}}} \end{aligned}$$
  9 $$\begin{aligned} {\mathfrak {S}^-}= & {\left( {\frac{1}{{2\mathfrak {n}}}\sum \limits _{\mathfrak {j} = 1}^\mathfrak {n}{{{\left( {\left( {\frac{{{{\hat{\gamma }}_\mathfrak {i}}}}{{{\zeta _\Lambda }({{\hat{\gamma }}_\mathfrak {i}})}},{\chi _\Lambda }({{\hat{\gamma }}_\mathfrak {i}})} \right) - \left( {\frac{{{{\hat{\gamma }}_\mathfrak {i}}}}{{\zeta _\Lambda ^-({{\hat{\gamma }}_\mathfrak {i}})}},\chi _\Lambda ^-({{\hat{\gamma }}_\mathfrak {i}})} \right) } \right) }^2}} } \right) ^{\frac{1}{2}}} \end{aligned}$$
- **Step 6:** Determine how near the positive and negative ideals answer you are in relation to it. The following defines the alternative $\Pi _{\Lambda }$’s relative closeness to the positive and negative ideals solution:
  10 $$\begin{aligned} {\mathfrak {C}_{{\mathfrak {i}^*}}} = \frac{{{\mathfrak {S}_{{\mathfrak {i}^ - }}}}}{{{\mathfrak {S}_{{\mathfrak {i}^*}}} + {\mathfrak {S}_{{\mathfrak {i}^ - }}}}} \end{aligned}$$
  where $0 \le {\mathfrak {C}_{{\mathfrak {i}^*}}} \le 1$ and $\mathfrak {i} = 1, 2,..., \mathfrak {m}$ are the values. The alternative is better evaluated when the index value is higher.
- **Step 7:** Sort the options according to your liking or select the one that comes closest to 1. This phase involves organizing a set of options according to the value of ${\mathfrak {C}_{{i^*}}}$, preferably in descending order.

#### Steps for implementing fuzzy parameterized fuzzy hypersoft set TOPSIS algorithm with entropy weights

- **Step:1 (Construct Decision Matrix)**
  The decision matrix is initially constructed using equation (1), and it is shown in Table A1.
- **Step 2: (Normalized Decision Matrix)**
  Normalizing the selected options is the second stage, as per equation (4.2) see (Table A2).
- **Step 3: (Weighted Normalized Matrix)**
  In this stage, the TOPSIS algorithm is integrated with the entropy weights, as shown in equation (4). Table A3 contains the resultant weighted normalized matrix.
- **Step 4: (Positive and Negative ideal Solutions)**
  This step involves determining the positive and negative ideal solutions using equations (6) and (7), which are shown in (Table A4 and A5) after the weights have been integrated.
- **Step 5: (Separation Measure)**
  Equations (8) and (9) provide the separation measure between positive and negative ideal solutions in this phase, which is shown in (Table A6).
- **Step 6 and 7: (Relative Closeness and Ranked the Alternative)**
  This phase involved ranking the options that are shown in (Table A7) and calculating the relative closeness using the equation (10).

### Fuzzy parameterized fuzzy hypersoft set MULTIMOORA method

The MULTI-MOORA method is divided into three separate approaches (i.e.,The Ratio System Approach, the Reference Point Approach, and the Full Multiplicative Form). The idea of dominance and the three methods used to rank the options under consideration are used to determine the final ranking and conclusion.

It basically states that the choice that appears the most on the top of all three lists of rankings is the one with the highest ranking. The step-by-step computation of information of each method is presented below:

- **Step 1: (The Ratio System Approach (RSA))**
  Using this approach, the general standing of the alternative $\mathfrak {i}$ can be ascertained as follows:
  11 $$\begin{aligned} {\mathfrak {y}_\mathfrak {i}} = \mathfrak {y}_\mathfrak {i}^ {+} - \mathfrak {y}_{\mathfrak {i}}^ {-} , \end{aligned}$$
  with,
  12 $$\begin{aligned} \mathfrak {y}_\mathfrak {i}^ {+} = \sum \limits _{\mathfrak {j} \in {\Upsilon _{\max }}} {{\mathfrak {w}_\mathfrak {j}}} {\mathfrak {r}_{\mathfrak {ij}}}, \end{aligned}$$
  and
  13 $$\begin{aligned} \mathfrak {y}_\mathfrak {i}^ {-} = \sum \limits _{\mathfrak {j} \in {\Upsilon _{\min }}} {{\mathfrak {w}_\mathfrak {j}}} {\mathfrak {r}_{\mathfrak {ij}}}, \end{aligned}$$
  where, $$\mathfrak {r}_\mathfrak {ij} ={{{\left( {\frac{{{{\hat{\gamma }}_\mathfrak {i}}}}{{{\zeta _\Lambda }({{\hat{\gamma }}_\mathfrak {i}})}},{\chi _\Lambda }({{\hat{\gamma }}_\mathfrak {i}})} \right) }}}$$ $$\mathfrak {i}=1,2,...,\mathfrak {m}; \mathfrak {j}=1,2,...,\mathfrak {n}$$
  14 $$\begin{aligned} \mathfrak {r}_\mathfrak {ij} = \frac{{{\Pi _\Lambda }}}{{\sqrt{\sum \limits _{\mathfrak {j} = 1}^\mathfrak {n} {{{({\Pi _\Lambda })}^2}} } }}, \end{aligned}$$
  where the ith position of the alternative is represented by $\mathfrak {y}_\mathfrak {i}$ based on all criteria; the ith positions of the alternative are represented by $\mathfrak {y}_\mathfrak {i}^{+}$ and $\mathfrak {y}_\mathfrak {i}^{-}$ based on benefit and cost criteria, respectively. $\Upsilon _{\max }$ shows the sets of benefit criteria, $\Upsilon _{\min }$ indicates the cost criteria, and $\mathfrak {r}_\mathfrak {ij}$ represents the normalized ith alternative under the jth criteria. $\Pi _\Lambda$ indicates the ith alternative related to the jth criterion. $\mathfrak {j} = 1,2,..., \mathfrak {n}$; $\mathfrak {i} = 1,2,..., \mathfrak {m}$. Since the related alternatives are organized based on $\mathfrak {y}_\mathfrak {i}$ in descending order, the option with the largest value of $\mathfrak {y}_\mathfrak {i}$ is the best one in this technique.
- **Step 2: (The Reference Point Approach (RPA))**
  The following technique could be used to select the best option:
  15 $$\begin{aligned} \mathfrak {d}_\mathfrak {i}^{\max } = \mathop {\max }\limits _\mathfrak {j} \left( {{\mathfrak {w}_\mathfrak {j}}\left| {\mathfrak {r}_\mathfrak {j}^* - {\mathfrak {r}_{\mathfrak {ij}}}} \right| } \right) \end{aligned}$$
  where $\mathfrak {d}_\mathfrak {i}^{\max }$ denotes the extreme distance of the alternative $\mathfrak {i}$ with respect to the reference point and $\mathfrak {r}_\mathfrak {j}^*$ represents the coordinate $\mathfrak {j}$ of the reference point:
  16 $$\begin{aligned} \mathfrak {r}_\mathfrak {j}^* = \left\{ {\begin{array}{*{20}{c}} {\mathop {\max }\limits _\mathfrak {i} {\mathfrak {r}_{\mathfrak {ij}}}, \;\;\; \mathfrak {j} \in {\Upsilon _{\max }}}\\ {\mathop {\min }\limits _\mathfrak {i} {\mathfrak {r}_{\mathfrak {ij}}}, \;\;\;\mathfrak {j} \in {\Upsilon _{\min }}} \end{array}} \right. \end{aligned}$$
  The $\mathfrak {d}_\mathfrak {i}^{\max }$ is sorted in ascending order to establish the final ranking of this method; the method with the lowest $\mathfrak {d}_\mathfrak {i}^{\max }$ value is considered the best.
- **Step 3: (The Full Form of Multiplication (FMF))**
  For this form, the total effectiveness of the alternative might be ascertained in the following way:
  17 $$\begin{aligned} {\mathfrak {u}_\mathfrak {i}} = \frac{{{\mathfrak {a}_\mathfrak {i}}}}{{{\mathfrak {b}_\mathfrak {i}}}}, \end{aligned}$$
  with
  18 $$\begin{aligned} {\mathfrak {a}_\mathfrak {i}}= & \prod \limits _{\mathfrak {j} \in {\Upsilon _{\max }}} {{\mathfrak {w}_\mathfrak {j}}} {\mathfrak {r}_{\mathfrak {ij}}}, \end{aligned}$$
  19 $$\begin{aligned} {\mathfrak {b}_\mathfrak {i}}= & \prod \limits _{\mathfrak {j} \in {\Upsilon _{\min }}} {{\mathfrak {w}_\mathfrak {j}}} {\mathfrak {r}_{\mathfrak {ij}}}. \end{aligned}$$
  In this instance, ${\mathfrak {u}_\mathfrak {i}}$ represents the ith alternative’s overall effectiveness, and $\mathfrak {b}_\mathfrak {i}$ and $\mathfrak {a}_\mathfrak {i}$ represent the product of the weighted performance ratings of the ith alternative’s cost and benefit criteria. In order to support RSA, the associated options are ranked using ${\mathfrak {u}_\mathfrak {i}}$ in decreasing order. The option with the highest ${\mathfrak {u}_\mathfrak {i}}$ value is selected as the best option.

#### Steps for implementing fuzzy parameterized fuzzy hypersoft set MULTIMOORA algorithm with entropy weights

Creating a decision matrix akin to TOPSIS is the first stage in this process, as shown in (Table A1). Three different methods are used by the MultiMOORA method to determine the ranks of the alternatives:

- **Step 1: (The Ratio System Approach)**
  This phase involves calculating the ratio system method using the data from Section 4.3 that is shown in (Table A8).
- ** Step:2 (The Reference Point Approach)**
  The reference point approach was computed in this stage using the data from Section 4.3 and is shown in (Table A9).
- ** Step:3 (The Full Multiplicative Form Approach)**
  The full multiplicative form was computed in this stage using the data from Section 4.3 and is shown in (Table A10).

## Simulation of tailored decision-making algorithms for optimal selection of wastewater treatment facility in various scenarios

In the changing world of wastewater treatment finding ways to be more efficient and environmentally friendly is a continuous challenge. Those in charge of making decisions have to consider many factors, such as the impact on the environment and society technical capabilities and economic feasibility. This complex task requires solutions that can offer clear and reliable guidance, for decision making processes. To address this multi-faceted data, this section focuses on applying FPFHSS approach to divide the attributes to sub-attributes for an in-depth analysis while also parameterized the data into fuzzy variables. It’s designed to handle the inherent uncertainties and imprecision that are prevalent in the waste management domain. By doing so, it equips decision-makers with a precise yet flexible tool to navigate this intricate landscape. Here, a step-by-step presentation of the application of the algorithm on the data is provided for the first case. For the sake of brevity, only the weights and the results of the second and the third cases are provided. Through the exploration of three distinct scenarios, we aim to showcase the versatility and effectiveness of this framework in real-world waste management decision-making.

### Case 1: Weight determination using the entropy method

There are several methods available for determining the weights of the criteria that are being considered, such as LINMAP, SMART, Eigenvector, expert survey method, Delphi method, and AHP. Subjective factors could make these processes laughable. Conversely, objective fixed weight techniques use naturally occurring indecisiveness data to establish criteria weights that reduce human error and yield more factually accurate results. Shannon introduced the concept of entropy, which may be used to evaluate the value and quantity of clutter in system data. The weights that are produced are known as objective fixed weights as this method is dependent on the provided facts to generate weights.

The concept of Shannon Entropy Weight has been studied in numerous domains, such as biological diversity, defect feature extraction, decision-making, attribute recognition, efficiency aggregation, and materials selection. For evaluating the quality of groundwater sources, Chen et al.^85^ developed an attribute recognition model based on entropy weight; in contrast, Guiaşu and Guiaşu^86^ investigated conditional and weighted measures of ecological diversity. For imprecise data in multi-attribute decision-making contexts, Lotfi and Fallahnejad^87^ extended the Shannon entropy technique. CRE stands for Cumulative Residual Entropy, a unique information measure introduced by Rao et al.^88^ that applies Shannon entropy to random variables with continuous distributions. A DEA cross-efficiency aggregation method based on Shannon entropy was proposed by Wu et al.^89^ to determine final cross-efficiency. The MULTIMOORA approach was extended by Hafezalkotob and Hafezalkotob^90^, who chose materials according to Shannon entropy weight. Sheng et al.^91^ presented a failure feature extraction method based on local mean decomposition Shannon entropy. Singh et al.^92^ introduced a Shannon entropy-based method for concept reduction in formal concept analysis using fuzzy features. All things considered, the application of Shannon entropy weight has proven effective in several contexts, providing a comprehensive approach for feature extraction, information evaluation, and decision-making in a variety of domains.

- **Step 1: (Normalization Matrix)**
  Index normalization according to (4.2).
- **Step 2: (Computation of the entropy index)**
  By definition, the following formula can be used to get the entropy of the jth criteria:
  20 $$\begin{aligned} {\mathfrak {e}_\mathfrak {j}} = - \mathfrak {h}\sum \limits _{\mathfrak {i} = 1}^\mathfrak {m} {{\mathfrak {r}_{\mathfrak {ij}}}} \ln {\mathfrak {r}_{\mathfrak {ij}}},(\mathfrak {i} = 1,2,...,\mathfrak {m};\mathfrak {j} = 1,2,...\mathfrak {n}). \end{aligned}$$
  Where,
  21 $$\begin{aligned} \mathfrak {h} = \frac{1}{{\ln (\mathfrak {m})}},(\mathfrak {i} = 1,2,...,\mathfrak {m};\mathfrak {j} = 1,2,...\mathfrak {n}) \end{aligned}$$
- **Step 3: (Computation of the entropy weight)**
  The entropy weight of the jth index can be determined by:
  22 $$\begin{aligned} { \mathfrak {w}_ \mathfrak {j}} = \frac{{1 - { \mathfrak {e}_ \mathfrak {j}}}}{{ \mathfrak {n} - \sum \limits _{ \mathfrak {i} = 1}^ \mathfrak {n} {{ \mathfrak {e}_ \mathfrak {j}}} }},\sum \limits _{ \mathfrak {i} = 1}^ \mathfrak {n} {{ \mathfrak {w}_ \mathfrak {j}}} = 1,( \mathfrak {j} = 1,2,..., \mathfrak {n}) \end{aligned}$$

#### Steps for Implementing Entropy Weights

- **Step 1: (Index Normalization)**
  Normalizing the selected options is the first stage, as per equation (4.2) see (Table A2).
- **Step 2: (Computation of Entropy Index)**
  Equations (20) and (21), which are shown in Tables A11 and A12, can be used to compute the entropy index:
- **Step 3: (Computation of Entropy Weight)**
  Table (2) displays the entropy weight of the jth index, which may be obtained using (22).

**Table 2:** Weights of Entropy for Attributes.

Alternatives/ Attributes	Environmental Factors			Social Factors				Technical Factors							Economic Factors				
	A1	A2	A3	A4	A5	A6	A7	A8	A9	A10	A11	A12	A13	A14	A15	A16	A17	A18	A19
Weights	0.059	0.166	0.049	0.0004	0.011	0.067	0.036	0.120	0.105	0.003	0.007	0.007	0.019	0.006	0.100	0.041	0.133	0.043	0.019

Following dataset analysis using the weights shown in the preceding table, the final ranking of alternatives using Table 2 weights is given below Table 3.

**Table 3:** Final Ranking of Alternatives for Case: 1.

Case 1																									
	FPFHSS					TOPSIS					MULTIMOORA														
											Ratio System Approach					Reference Point Approach					Full Multiplicative Form				
	Score Values		Order		Ranking	Score Values		Order		Ranking	Score Values		Order		Ranking	Score Values		Order		Ranking	Score Values		Order		Ranking
Alt 1	0.806917304		A3>A4>A1>A2		3	0.225572506		A3>A4>A1>A2		3	0.049551267		A3>A4>A1>A2		3	0.037344946		A3>A4>A1>A2		3	240.0571807		A3>A4>A1>A2		3
Alt 2	0.702396087				4	0.166587901				4	0.035931944				4	0.032649519				4	181.728221				4
Alt 3	0.260135764				1	0.882855252				1	0.161820189				1	0.134987937				1	762.2718146				1
Alt 4	0.443034734				2	0.241969116				2	0.059590459				2	0.051850884				2	316.6760351				2

### Case 2: Equal Weightage for social, technical, environmental, and economic factors

In situations where decisions need to be made stakeholders often perceive all aspects of wastewater management as equally significant. This approach ensures that a fair and thorough decision making process takes into account all factors involved. When all four factors carry importance decision makers are faced with the complex task of striking a balance between diverse and sometimes conflicting elements of waste management. Social considerations may pertain to the well being of the community. How the public perceives it while technical factors encompass the practical capabilities of waste management technologies. Environmental aspects focus on sustainability and the impact on ecology while economic factors revolve around cost effectiveness. FPFHSS excels, in achieving this balance ensuring that no aspect is overlooked and that all dimensions are evaluated impartially.

The initial steps involve defining alternatives, categorizing attributes, and creating a comprehensive evaluation set for systematic decision-making. In this particular scenario, a hypothetical near to real data set is used for explaining the functionality of the presented approach. The process starts by defining the initial set of alternatives represented by $\acute{B}$ and a set of attributes represented by $\mathbb {E}$. In the wastewater treatment system example, $\acute{B}$ would consist of the wastewater treatment systems, and $\mathbb {E}$ would encompass the criteria that are being evaluated, such as Environmental Factors, Social Factors, Technical Factors, and Economic Factors. These attributes are then organized into distinct, non-overlapping sub-classes based on sub-parametric values. For example, the ”Environmental Factors” criterion can be categorized as $\bigg \{A_{1}, A_{2}, A_{3}\bigg \}$,”Social Factors” as $\bigg \{A_{4}, A_{5}, A_{6}, A_{7}\bigg \}$, ”Technical Factors” as $\bigg \{A_{8}, A_{9}, A_{10}, A_{11}, A_{12}, A_{13}, A_{14}\bigg \}$, and ”Economic Factors” as $\bigg \{A_{15}, A_{16}, A_{17}, A_{18}, A_{19}\bigg \}$. Next, calculate the set $\Upsilon$, which represents the product of the subsets $\bigg \{A_{1}, A_{2}, A_{3}, \ldots , A_{19}\bigg \}$. This stage basically entails merging the criteria to produce the decision matrix, which is an extensive collection of attributes for assessment. Table A13 presents the information described.

The next step is to parameterize the qualities into fuzzy membership values after the decision matrix has been formed. As there are 4 attributes which are further divided into 19 sub-attributes each having different nature of data in them (i.e., linguistic, crisp, interval based etc) as highlighted in Table 1, specialized scales need to be developed for each of them for proper fuzzy parameterization. Based on the diverse nature of the data, the constructed parameterization guides for environmental factors are presented in Table A14, social factors in Table A15, technical factors in A16, and economic factors in Table A17. Following a cross-reference with the created parameterization scales, fuzzy membership values are assigned to the decision matrix values, resulting in a transformation of the original matrix with values having fuzzy memberships. The transformed decision matrix is presented in Table A1.

Next, consider a set $\mathfrak {H}$, which is a subset of $\Upsilon$. The selection of elements within $\mathfrak {H}$ should not surpass a specific limit, a determination reached through consultations with decision-makers are as follows (Table A1):

Next, construct the $\Lambda$-set, denoted as $\Pi _{\Lambda }$, following the principles outlined in Definition 3. For each element in the original set $\acute{B}$, compute ${\aleph _\Lambda ^U}$ using the instructions detailed in Definition 4. This stage entails assessing each alternative based on the criteria and their respective weights. The determination of $\Pi _\Lambda ^U$ aligns with the directions provided in Definition 4, which encompass aggregating the evaluations of each alternative to ascertain their overall performance are presented in Table 4 and A18.

**Table 4:** Weight Assignment and Criteria Set Formation.

Alternatives/Attributes	Environmental Factors			Social Factors				Technical Factors							Economic Factors				
	A1	A2	A3	A4	A5	A6	A7	A8	A9	A10	A11	A12	A13	A14	A15	A16	A17	A18	A19
Weights	0.0833	0.0833	0.0833	0.0625	0.0625	0.0625	0.0625	0.0357	0.0357	0.0357	0.0357	0.0357	0.0357	0.03571429	0.05	0.05	0.05	0.05	0.05

As seen in Table 4, Table A18 gives information on the fuzzy parameterized set attributive set values together with the relative relevance of each alternative.

Following the creation of the parameterized sets, the score value of $\Pi _{_\Lambda }^{{U_{1}}}$ must be ascertained using the criteria given in Definition (3.4). Finally, select the option with the highest value as the final choice from the set ${\aleph _\Lambda ^U}(\mathbb {a}_i)$. This relates to the wastewater treatment system that, when considered in the context of Table 5, exhibits the best performance in light of the weighted criteria.

**Table 5:** Final Ranking of Alternatives for Case: 2.

Case 2																									
	FPFHSS					TOPSIS					MULTIMOORA														
											Ratio System Approach					Reference Point Approach					Full Multiplicative Form				
	Score Values		Order		Ranking	Score Values		Order		Ranking	Score Values		Order		Ranking	Score Values		Order		Ranking	Score Values		Order		Ranking
Alt 1	0.014119575		A3>A1>A4>A2		3	0.329941406		A3>A1>A4>A2		3	0.051291956		A3>A1>A4>A2		2	0.048988514		A3>A1>A4>A2		2	8.032166737		A4>A2>A3>A1		4
Alt 2	0.012108033				4	0.267303021				4	0.03043334				4	0.030297002				4	10.32971331				2
Alt 3	0.02539566				1	0.74303216				1	0.074156933				1	0.071334759				1	8.458897455				3
Alt 4	0.014830431				2	0.366614935				2	0.046295987				3	0.047330857				3	14.04586842				1

### Case 3: Emphasizing environmental factors

Environmental sustainability is a paramount concern in waste management today. Many environmental agencies and organizations prioritize minimizing the ecological impact of waste disposal. FPFHSS allows us to emulate such environmental policies effectively. By assigning higher weights to environmental factors in our decision-making process, we ensure that waste management plant selection aligns with the objectives of environmental protection. This scenario highlights the adaptability of FPFHSS to accommodate different policy-driven emphases that are represented in Table 6 and 7.

**Table 6:** Weight Assignment and Criteria Set Formation.

Alternatives/Attributes	Environmental Factors			Social Factors				Technical Factors							Economic Factors				
	A1	A2	A3	A4	A5	A6	A7	A8	A9	A10	A11	A12	A13	A14	A15	A16	A17	A18	A19
Weights	0.15	0.15	0.15	0.025	0.025	0.025	0.025	0.05	0.05	0.05	0.05	0.05	0.05	0.05	0.02	0.02	0.02	0.02	0.02

**Table 7:** Final Ranking of Alternatives for Case: 3.

Case 3																									
	FPFHSS					TOPSIS					MULTIMOORA														
											Ratio System Approach					Reference Point Approach					Full Multiplicative Form				
	Score Values		Order		Ranking	Score Values		Order		Ranking	Score Values		Order		Ranking	Score Values		Order		Ranking	Score Values		Order		Ranking
Alt 1	0.017075346		A3>A1>A4>A2		2	0.385651469		A3>A1>A4>A2		3	0.097932212		A3>A1>A4>A2		2	0.085610938		A3>A1>A4>A2		2	36.14475032		A4>A2>A3>A1		4
Alt 2	0.011483102				4	0.205915796				4	0.058560878				4	0.045272051				4	33.19426084				2
Alt 3	0.026243213				1	0.681024834				1	0.132467271				1	0.123774663				1	11.0735728				3
Alt 4	0.016077008				3	0.387816327				2	0.090179611				3	0.076618192				3	59.31607088				1

### Case 4: Prioritizing technical and economic factors for long-term sustainability

In pursuit of maximum sustainability and long-term utility, there are situations where technical and economic considerations take precedence over others. FPFHSS is well-suited to address this scenario by allowing decision-makers to assign higher weights to technical and economic factors. This approach ensures that the selected waste management plant not only meets immediate needs but also provides long-term benefits in terms of efficiency and cost-effectiveness. Based on that, in this case, the variation of weights that aligns with the broader goal of sustainable waste management practices that represented in Table 8 and 9.

**Table 8:** Weight Assignment and Criteria Set Formation.

Alternatives/Attributes	Environmental Factors			Social Factors				Technical Factors							Economic Factors				
	A1	A2	A3	A4	A5	A6	A7	A8	A9	A10	A11	A12	A13	A14	A15	A16	A17	A18	A19
Weight	0.05	0.05	0.05	0.0375	0.0375	0.0375	0.0375	0.05	0.05	0.05	0.05	0.05	0.05	0.05	0.07	0.07	0.07	0.07	0.07

After analysis of the dataset by using the weights presented in the above table, the final ranking of alternatives based on the weights provided in Table 8 is provided below 9:

**Table 9:** Final Ranking of Alternatives for Case: 4.

Case 4																									
	FPFHSS					TOPSIS					MULTIMOORA														
											Ratio System Approach					Reference Point Approach					Full Multiplicative Form				
	Score Values		Order		Ranking	Score Values		Order		Ranking	Score Values		Order		Ranking	Score Values		Order		Ranking	Score Values		Order		Ranking
Alt 1	0.013342659		A3>A4>A1>A2		3	0.270141397		A3>A4>A2>A1		4	0.029393109		A3>A4>A2>A1		4	0.031894271		A3>A4>A2>A1		4	8.032166737		A3>A2>A4>A1		4
Alt 2	0.012785042				4	0.274187527				3	0.035455488				3	0.03588263				3	10.37154836				2
Alt 3	0.026521607				1	0.824810462				1	0.058255186				1	0.059513876				1	11.152121				1
Alt 4	0.015017452				2	0.343651664				2	0.036370389				2	0.037637896				2	8.160585547				3

## Results and discussion

**Fig. 3:**
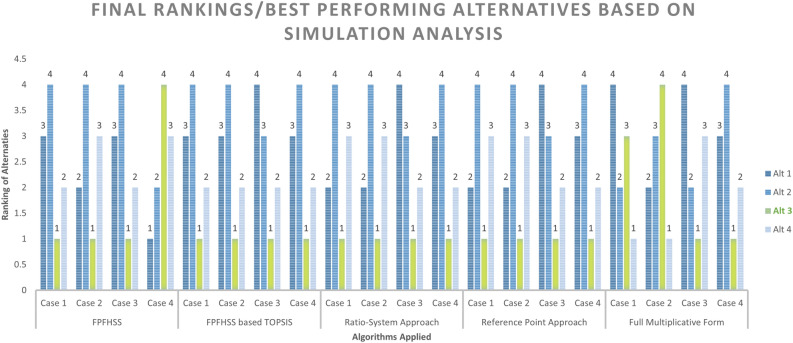
Graphical Representation of Rankings obtained from the Simulation Analysis.

The decision-making approach highlighted in the provided information advocates for an equitable consideration of social, technical, environmental, and economic factors in waste management. This balanced method begins by defining alternatives and criteria, categorizing them, and consulting with decision-makers to set limits on the number of criteria. The relative importance of each criterion is then represented by a weight, and the fuzzy parameterization method is used to assess the alternatives in light of the weighted criteria. In the context of waste management, we conduct a comprehensive analysis of four distinct alternatives, denoted as Alt 1, Alt 2, Alt 3, and Alt 4, across four distinct scenarios, referred to as 5.1(Case 1), 5.2(Case 2), 5.3(Case 3), and 5.4(Case 4). The graphical and Tabular representation of the results is presented in Figure 3 and Table 10 respectively.

In **Case 1**, weights are calculated by entropy method. Because it is an objective technique based on the inherent qualities of the data, the entropy weights measure has an advantage over subjective weighting approaches in that it minimizes bias. It guarantees that the decision-making process is more influenced by criteria that are more informative. In this scenario, Table 3 presents the final ranking of four alternatives (Alt 1, Alt 2, Alt 3, and Alt 4) using three different multi-criteria decision-making methods: FPFHSS, TOPSIS, and MULTIMOORA, it encompasses the full multiplicative form, reference point approach, and ratio system approach. Each method consistently ranked the alternatives in the same order: **Alt 3** as the top choice, followed by **Alt 4**, **Alt 1**, and **Alt 2**. This uniform ranking across all methods indicates a robust decision-making framework, where **Alt 3** consistently outperforms the other alternatives, suggesting high confidence in selecting it as the best option. The alignment in rankings underscores the effectiveness of the chosen criteria and weights, ensuring a decisive and well-supported decision-making process.

In **Case 2**, weights of the major factors (i.e., environmental, social, technical, and economic) are all kept equal emulating a scenario where the importance of each factor is equal. In this scenario, Table 5 presents the final ranking of alternatives for Case 2 using three decision-making methods: FPFHSS, TOPSIS, and MULTIMOORA. FPFHSS and TOPSIS rank alternatives primarily based on score values, with **Alt 3** consistently leading in both methods, followed by **Alt 4** and then **Alt 1** and **Alt 2**. Three different strategies are used by MULTIMOORA: the ratio system, reference point, and full multiplicative form. While **Alt 3** is the clear winner in the reference point approach, **Alt 4** holds the top spot in the ratio system. On the other hand, **Alt 2** secures the second place with remarkable performance in the full multiplicative form. All things considered, **Alt 3** is the most effective choice when compared to other approaches. **Alt 4** is strong when it comes to the ratio system, and **Alt 2** performs admirably when it comes to the full multiplicative form. Using every technique, **Alt 1** and **Alt 2** consistently rank lower.

Transitioning to **Case 3** where greater importance is given to factors associated with the environment, evaluating alternatives using FPFHSS, TOPSIS, and various approaches within MULTIMOORA. The final rankings, depicted in 7, reveal varying assessments across methods. While FPFHSS and TOPSIS both rank **Alt 3** highest, MULTIMOORA’s different approaches yield divergent results, with **Alt 3** ranked highest in the ratio system and reference point approaches, and **Alt 4** ranked highest in the full multiplicative form.

In the **Case 4**, FPFHSS approach enables prioritization of technical and economic factors for maximum sustainability and long-term utility in waste management plant selection, ensuring efficiency and cost-effectiveness. Table 9 presents the final rankings of alternatives for Case 4, utilizing FPFHSS, TOPSIS, and various methods within MULTIMOORA. Across the methods, **Alt 3** consistently emerges as the top-ranked alternative, indicating its superior performance compared to the other alternatives. FPFHSS and TOPSIS both rank **Alt 3** highest, while within MULTIMOORA, **Alt 3** is also ranked highest across all three approaches: ratio system, reference point, and full multiplicative form. Conversely, **Alt 2** consistently ranks lowest across all methods, suggesting its comparatively poorer performance. The rankings highlight the robustness of **Alt 3’s** performance across different decision-making methodologies, indicating its strength as a preferred alternative in Case 4. This consistency underscores the importance of employing multiple evaluation techniques to ensure a comprehensive and reliable assessment of alternatives in decision-making processes. The final rankings are also presented in tabulated from below:

**Table 10:** Final Rankings from all cases.

Alternatives	FPFHSS				FPFHSS based TOPSIS				FPFHSS based Multi-MOORA										
									Ratio-System Approach				Reference Point Approach				Full Multiplicative Form		
	Case 1	Case 2	Case 3	Case 4	Case 1	Case 2	Case 3	Case 4	Case 1	Case 2	Case 3	Case 4	Case 1	Case 2	Case 3	Case 4	Case 1	Case 3	Case 4
Alt 1	3	2	3	1	3	3	4	3	2	2	4	3	2	2	4	3	4	4	3
Alt 2	4	4	4	2	4	4	3	4	4	4	3	4	4	4	3	4	2	2	4
Alt 3	1	1	1	4	1	1	1	1	1	1	1	1	1	1	1	1	3	1	1
Alt 4	2	3	2	3	2	2	2	2	3	3	2	2	3	3	2	2	1	3	2

In this research, social, technical, environmental and economic concerns are all used equally to find sustainable answers to waste problems. To start, the process sorts out the criteria and alternatives to find the area where a decision can be made and then it consults the responsible group to find out the practical number of criteria to be considered. For each objective, a score shows the importance and the alternatives are evaluated by how well they meet those requirements. As a consequence, different sides of the issue can be considered, by checking both what the policy tries to achieve and what the local community faces. Its power lies in the fact that the framework adjusts to new challenges while keeping the same set of promised alternatives (such as Alt 3) which can be relied on and repeated for sustainable development.

This approach ensures that the decision-making process in waste management is comprehensive and well-rounded, taking into account various critical factors and adapting to different scenarios. Alt 3 consistently performs well in terms of score values and rankings, suggesting its suitability in multiple waste management contexts, while Alt 2 exhibits less dynamic performance across the scenarios. Alt 1 and Alt 4 show adaptability to varying circumstances but display fluctuations in their performance metrics as illustrated in Table 3, 5, 7, 9. The fluctuation of weights also provides great insight in terms of expression of sensitivity of attributive values. By greatly varying the weights as illustrated in each case, the highest ranking alternative remains the same throughout while some variation is observed in the ranking of the other three alternatives. Based on our findings, in order for improved monitoring and control of the parameters, the government must develop standards and regulations when computing sustainable measures^93^. Among all the factors, we found that sludge was one of the leading factors that had major effects on the energy cost, operational and maintenance of the wastewater treatment facilities^94^.

The presented results and discussions offer several distinct advantages, including a comprehensive and systematic approach to waste management decision-making method that encompasses social, technical, environmental, and economic factors, promoting a holistic assessment of alternatives. In terms of the managerial implications of the proposed approach, the hybrid of hypersoft set and fuzzy parameterization not only allows for a detailed analysis up to a sub-attributive level but also handles uncertainty and incomplete data better, making it highly suitable for environmental decision-making applications where the data is uncertain and incomplete. By replacing the thresholds by graded membership values, it allows managers to incorporate a spectrum of uncertainties when analyzing environmental data. Also, the cases of conflicting criteria such as safety, sustainability, and cost are better handled and balanced by these computational systems as these models are systematic in their approach and completely free of any subjective behavior. This allows managers to expertly handle trade-off scenarios and prioritize objectives of competing nature based on a transparent aggregation system. The methodology’s adaptability to different policy priorities, transparent evaluation process, and versatility in alternative selection ensure that it can effectively align with evolving environmental protection objectives and economic considerations while allowing for clear communication of the reasoning behind chosen waste management solutions. Furthermore, the emphasis on long-term sustainability emphasizes the need to optimize efficiency and economic viability for long-term waste management procedures. The approach’s interdisciplinary promotes collaboration among specialists from other professions, resulting in more robust conclusions, and its consideration of social ramifications improves community well-being and public acceptance. Its global application potential and forward-thinking approach make it a powerful tool for tackling shifting environmental issues and technological breakthroughs while retaining transparency and accountability in decision-making.

### Comparative analysis

Numerous uses of fuzzy parameterized tools for the analysis of diverse decision-making situations have been documented in the literature. Based on the set structure used to address the problem, the degree of addressing characteristics, the type of data sets utilized for analysis, and the application of criteria for fuzzification of fuzzy parameters, Table 11 compares the findings of other studies. Fuzzy Parameterized Fuzzy Soft-set (FPFS-set) and Riesz Summability approach, which were presented by Caugman et al.^95^ and Altay et al.^96^, respectively, were compared in a study by Yilmaz et al.^97^. Their hypothetical data only addressed surface-level attributes, and they used it to solve decision-making issues in an unpredictable setting. More uncertainty handling was also claimed to be possible with fuzzy parameterization, particularly when there were several kinds of parameters. Kiricsci et al.^98,99^ used fuzzy soft set-based decision-making approaches to construct a tool to assist in the detection of heart disease using a Cardiovascular Dataset (CD-set). As a generalization of Fuzzy Soft Set^100^ and fpfs-set^95,101,102^, $\Lambda -set$ was introduced by Rahman et al.^103^). The study utilized $\Lambda -set$ to answer medical decision-making problems with real attribute values from a CD-set by utilizing fuzzy decision set procedures, which are a variation of aggregations from Caugman et al.^95^. For 11 of the 14 required qualities from the CD-set, Kirişci used a single-argument approximation function of fs-set, generating hypothetical fuzzy membership values in the absence of a defined criterion.

**Table 11:** Comparing the presented structure with pre-existing approaches through a structural analysis.

Authors		Structures		Focus on Attributes			Focus on sub attributive values			Data Set		Proper Criteria for Fuzzification of Fuzzy Parameters		
Çağman et al.^95^		FPFS-set		$\checkmark$			$\times$			Hypothetical		$\times$		
Yılmaz and Eraslan^97^		FPFS-set		$\checkmark$			$\times$			Hypothetical		$\times$		
Kirişci^98,99^		FPFS-set		$\checkmark$			$\times$			CD-set		$\times$		
Riaz and Hashmi^102^		FPFS-set		$\checkmark$			$\times$			Hypothetical		$\times$		
Zhu and Zhan^101^		FPFS-set		$\checkmark$			$\times$			Hypothetical		$\times$		
Rahman et al.^81^		FPFHSS		$\checkmark$			$\checkmark$			Hypothetical		$\times$		
Rahman et al.^104^		FPFHSS		$\checkmark$			$\checkmark$			CD-set		$\checkmark$		
Proposed Study		FPFHSS		$\checkmark$			$\checkmark$			Pseudo-realistic		$\checkmark$		

However, this approach raises concerns about the reliability of decision-making, as it overlooks sub-parametric values of adopted attributes. Specifically, the existing models mentioned above lack the ability to collectively handle two critical scenarios. Firstly, they struggle in situations where parameters and their tuples based on sub-parametric values are ambiguous, leading to uncertainty among decision-makers regarding their preference-based selection. Secondly, these models face challenges in situations requiring the categorization of parameters into distinct subclasses with associated sub-parametric values. A multi-argument approximation function that can handle sub-parametric-valued disjoint classes is required to solve this. This function’s range is made up of subsets of the original universe, and its domain is the Cartesian product of these classes. Upon comparison, the results have proven to be successful, and it has been noted that the methodology’s adaptability and comprehensive approach make it well-suited for diverse structures, providing a transparent and versatile framework for waste management decision-making. Its interdisciplinary nature fosters collaboration, ensuring robust decisions, while the emphasis on long-term sustainability and consideration of social implications enhance community well-being and global applicability.

### Limitations of the study

The presented results come with certain limitations. These include potential challenges arising from data availability and quality, subjectivity in weight assignment, sensitivity to parameter selection, the computational complexity and resource requirements of the FPFHSS methodology, and the need for improved methods for community engagement. Furthermore, the methodology’s applicability in various cultural and geographical contexts remains incompletely studied, and it may not sufficiently account for dynamic alterations in environmental elements. Further factors that might affect the viability of selected waste management solutions and necessitate further careful thought are the larger political and economic backdrop, as well as the changing nature of waste management practices. These drawbacks highlight the necessity of constant improvement and modification of the strategy in order to successfully handle these complications.

### Potential applications and future directions

The potential applications of a fuzzy parameterized fuzzy hypersoft set are vast. It could be used in domains such as risk assessment, where it can model complex risk scenarios incorporating imprecise data and advanced uncertainty handling. By taking individual differences in medical data into account, it could improve the accuracy of medical diagnoses. This idea may be used in natural language processing to represent the complex and context-dependent nature of spoken discourse. Environmental modeling could make use of decision support systems’ capacity to manage contradicting data while taking user preferences into account to produce more resilient and adaptable models in the face of environmental unpredictability. The idea does, however, also offer a number of difficulties and chances for additional study. It is imperative to provide a rigorous mathematical formalism that characterizes the interactions between variables inside a hypersoft computing environment. Managing computational complexity in large-scale or real-time systems and effectively integrating inaccurate data from several sources are major problems.

Also, an interdisciplinary collaboration among mathematicians, computer scientists, domain experts, and engineers is essential to realize the full potential of a fuzzy parameterized fuzzy hypersoft set. With these advantages supporting the applications of the structure, this concept represents a cutting-edge approach to managing complex uncertainty and holds great promise across diverse fields, but its practical development and application require further exploration and collaboration. The future direction of this research involves harnessing emerging technologies for more precise and efficient waste management decision-making, refining sustainability metrics to address evolving environmental concerns, engaging communities and stakeholders to ensure holistic solutions, developing specific policy frameworks to align with the FPFHSS methodology, establishing long-term performance monitoring systems for continuous improvement, exploring global applications of the approach in various contexts, and fostering interdisciplinary collaboration to enhance the comprehensiveness and effectiveness of waste management practices.

## Conclusion

In the ever-evolving urban landscape, the pursuit of sustainability necessitates a comprehensive, multifaceted approach incorporating social, economic, technical, and environmental considerations. This research presents a dynamic and hybrid Fuzzy Parameterized Fuzzy Hypersoft Set (FPFHSS) decision-making tool to be used as a comprehensive performance evaluation system for sustainable urban development, which in case was performance analysis of wastewater treatment systems. A distinctive feature of FPFHSS, the hypersoft structure, has allowed us to break down complex attributes into more manageable sub-attributes, while the fuzzy parameterization aspect allows for better handling of uncertainty and imprecise information. To further enhance the analysis, we have also developed a novel specialized FPFHSS based TOPSIS and MULTIMOORA approaches to provide a versatile analysis as each algorithm provides situational results. To further demonstrate the versatility and utility of FPFHSS, we have presented four distinct case studies, each focusing on four primary attributes further subdivided into 19 sub-attributes. These case studies, which prioritize different attributes, effectively emulate various scenarios encountered in urban development. As such, they serve as a comprehensive guide to informed and efficient decision-making. Some limitations of the proposed methodology include challenges arising from customized data availability and quality, subjectivity in weight assignment, and sensitivity to parameter selection. The future directions of FPFHSS include design of specialized risk assessment, forecasting, and modelling algorithms, where it can address complex risk scenarios incorporating imprecise data and advanced uncertainty handling to be applied in the fields of medical science, environmental modelling, and real-time monitoring systems for decision-making applications.

## Supplementary Information

Supplementary Information.

## Data Availability

All data generated or analysed during this study are included in this published article.
