# Supplementary material for: A decision-theoretic framework for wastewater treatment performance assessment based on a fuzzy parameterized fuzzy hypersoft set approach
Source: Sci Rep. 2025 Jul 1;15:20706. doi: 10.1038/s41598-025-07896-5 (PMC12217879; doi:10.1038/s41598-025-07896-5)
Supplement: Supplementary file 1 — Supplementary Information. [file 41598_2025_7896_MOESM1_ESM.pdf]

A Supplementary Material

	Environmental Factors			Social Factors				Technical Factors							Economic Factors				
Alternatives/Attributes	A1	A2	A3	A4	A5	A6	A7	A8	A9	A10	A11	A12	A13	A14	A15	A16	A17	A18	A19
Alt 1	0.8	0.2	0.7	0.8	0.4	0.1	0.5	0.2	0.3	1	0.9	0.9	0.5	0.8	0.2	0.2	0.5	0.2	0.6
Alt 2	0.5	0.1	0.3	0.8	0.4	0.5	0.3	0.3	0.1	0.9	0.9	0.9	0.6	0.5	0.1	0.7	0.2	0.6	0.7
Alt 3	0.1	0.9	1	0.6	0.5	0.5	0.6	0.7	0.7	0.9	1	1	0.8	0.5	0.6	0.6	0.9	0.6	0.9
Alt 4	0.7	0.2	0.8	0.7	0.5	0.3	0.3	0.1	0.3	1	0.9	0.9	0.6	0.7	0.6	0.5	0.1	0.4	0.6

Table A1. Fuzzy Parameterization of the attributive values presented in Table A13

	Environmental Factors			Social Factors				Technical Factors							Economic Factors				
Alternatives/ Attributes	A1	A2	A3	A4	A5	A6	A7	A8	A9	A10	A11	A12	A13	A14	A15	A16	A17	A18	A19
Alt 1	0.671	0.157	0.395	0.512	0.391	0.111	0.476	0.195	0.284	0.479	0.433	0.433	0.339	0.585	0.189	0.166	0.371	0.181	0.364
Alt 2	0.402	0.075	0.162	0.491	0.375	0.534	0.273	0.280	0.090	0.414	0.416	0.416	0.390	0.350	0.090	0.560	0.142	0.521	0.407
Alt 3	0.114	0.970	0.772	0.525	0.668	0.762	0.781	0.934	0.907	0.590	0.659	0.659	0.742	0.500	0.777	0.684	0.914	0.743	0.746
Alt 4	0.611	0.164	0.470	0.467	0.508	0.348	0.297	0.101	0.295	0.499	0.451	0.451	0.424	0.533	0.592	0.434	0.077	0.377	0.378

Table A2. Normalized Decision Matrix

	Environmental Factors			Social Factors				Technical Factors							Economic Factors				
Alternatives/ Attributes	A1	A2	A3	A4	A5	A6	A7	A8	A9	A10	A11	A12	A13	A14	A15	A16	A17	A18	A19
Alt 1	0.039	0.026	0.019	0.0002	0.004	0.007	0.017	0.023	0.029	0.001	0.003	0.003	0.006	0.003	0.019	0.006	0.049	0.007	0.007
Alt 2	0.023	0.012	0.008	0.0001	0.004	0.036	0.009	0.033	0.009	0.001	0.003	0.003	0.007	0.002	0.009	0.023	0.019	0.022	0.007
Alt 3	0.006	0.162	0.038	0.0002	0.007	0.051	0.028	0.112	0.095	0.001	0.004	0.004	0.014	0.003	0.078	0.028	0.122	0.032	0.014
Alt 4	0.036	0.027	0.023	0.0001	0.005	0.023	0.010	0.012	0.031	0.001	0.003	0.003	0.008	0.003	0.059	0.017	0.010	0.016	0.007

Table A3. Weighted Normalized Matrix

Positive Ideal	Environmental Factors			Social Factors				Technical Factors							Economic Factors				
	A1	A2	A3	A4	A5	A6	A7	A8	A9	A10	A11	A12	A13	A14	A15	A16	A17	A18	A19
	0.039	0.162	0.038	0.0002	0.007	0.051	0.028	0.112	0.095	0.001	0.004	0.004	0.014	0.003	0.078	0.028	0.122	0.032	0.014

Table A4. Positive Ideal Solution

Negative Ideal	Environmental Factors			Social Factors				Technical Factors							Economic Factors				
	A1	A2	A3	A4	A5	A6	A7	A8	A9	A10	A11	A12	A13	A14	A15	A16	A17	A18	A19
	0.006	0.012	0.008	0.0001	0.004	0.007	0.009	0.012	0.009	0.001	0.003	0.003	0.006	0.002	0.009	0.006	0.010	0.007	0.007

Table A5. Negative Ideal Solution

	Positive Ideal	Negative Ideal
Alt 1	0.03458133	0.010073
Alt 2	0.03850333	0.007696
Alt 3	0.00548539	0.04134
Alt 4	0.03615013	0.011539

Table A6. Separation Measure

	Relative Closeness	Rank
Alt 1	0.22557251	3
Alt 2	0.1665879	4
Alt 3	0.88285525	1
Alt 4	0.24196912	2

Table A7. Separation Measure and Ranked the alternatives

Ratio System Approach					
$\mathfrak{Y}_i^+$	Max	$\mathfrak{Y}_i^-$	Min	$\mathfrak{Y}_i$	Rank
Alt 1	0.04975854	Alt 1	0.00020728	0.04955127	3
Alt 2	0.03613076	Alt 2	0.00019882	0.03593194	4
Alt 3	0.16203275	Alt 3	0.00021257	0.16182019	1
Alt 4	0.05977923	Alt 4	0.00018877	0.05959046	2

Table A8. Ratio System Approach

Reference Point Approach		
	$\mathfrak{d}_i^{max}$	Rank
Alt 1	0.03734495	3
Alt 2	0.03264952	4
Alt 3	0.13498794	1
Alt 4	0.05185088	2

Table A9. Reference Point Approach

Full Multiplicative Form		
	Score Value	Rank
Alt 1	240.057181	3
Alt 2	181.728221	4
Alt 3	762.271815	1
Alt 4	316.676035	2

Table A10. Full Multiplicative Form

	Environmental Factors			Social Factors				Technical Factors							Economic Factors				
Alternatives/ Attributes	A1	A2	A3	A4	A5	A6	A7	A8	A9	A10	A11	A12	A13	A14	A15	A16	A17	A18	A19
	0.853	0.585	0.877	0.998	0.972	0.832	0.910	0.700	0.738	0.992	0.981	0.981	0.951	0.983	0.749	0.897	0.667	0.892	0.951

Table A11. Calculate Entropy Index by Using (20)

	Environmental Factors			Social Factors				Technical Factors							Economic Factors				
Alternatives/ Attributes	A1	A2	A3	A4	A5	A6	A7	A8	A9	A10	A11	A12	A13	A14	A15	A16	A17	A18	A19
	0.146	0.414	0.122	0.001	0.027	0.167	0.089	0.299	0.261	0.007	0.018	0.018	0.048	0.016	0.250	0.102	0.332	0.107	0.048

Table A12. Calculate Entropy Index by Using (21)

	Environmental Factors			Social Factors				Technical Factors							Economic Factors				
Alternatives/Attributes	A1	A2	A3	A4	A5	A6	A7	A8	A9	A10	A11	A12	A13	A14	A15	A16	A17	A18	A19
Alt 1	533	1683	107218	2	72	1	5	2	3	93	85	84	11	16	64	573990	7122	24	111689
Alt 2	596	1744	86734	2	68	5	3	3	1	84	83	86	12	10	42	268932	8712	14	80280
Alt 3	666	735	123015	4	78	5	6	7	7	82	95	95	16	11	129	293200	5237	15	47143
Alt 4	547	1634	112400	3	73	3	3	1	3	92	83	83	13	15	121	375581	9059	19	118841

Table A13. Explanation of sub-parametric values associated with selected parameters

Environmental Factors								
Energy Consumption (MW/Year)			Sludge Generation (Tonn/Year)			Water Reuse (Litre/Year)		
Lower Bound	Upper Bound	Membership Value	Lower Bound	Upper Bound	Membership Value	Lower Bound	Upper Bound	Membership Value
0	499	1	0	699	1	0	75000	0
500	520	0.9	700	830	0.9	75000	80000	0.1
521	540	0.8	831	960	0.8	80001	85000	0.2
541	560	0.7	961	1090	0.7	85001	90000	0.3
561	580	0.6	1091	1220	0.6	90001	95000	0.4
581	600	0.5	1221	1350	0.5	95001	100000	0.5
601	620	0.4	1351	1480	0.4	100001	105000	0.6
621	640	0.3	1481	1610	0.3	105001	110000	0.7
641	660	0.2	1611	1740	0.2	110001	115000	0.8
661	680	0.1	1741	1870	0.1	115001	120000	0.9
681	700	0	1871	2000	0	120001	125000	1

Table A14. Fuzzy Parameterization Scales for Environmental Factors

Social Factors											
Odor Problem (reference scale)			Manpower Requirement (Job Creation)			Social Acceptance (Scaled)			Social Benefits (Scaled)		
Lower	Upper	Membership	Lower	Upper	Membership	Lower	Upper	Membership	Lower	Upper	Membership
0	0	1	0	0	0	0	0	0	0	0	0
1	1.9	0.9	20	33	0.1	1	1.9	0.1	1	1.9	0.1
2	2.9	0.8	34	46	0.2	2	2.9	0.2	2	2.9	0.2
3	3.9	0.7	47	59	0.3	3	3.9	0.3	3	3.9	0.3
4	4.9	0.6	60	72	0.4	4	4.9	0.4	4	4.9	0.4
5	5.9	0.5	73	85	0.5	5	5.9	0.5	5	5.9	0.5
6	6.9	0.4	86	98	0.6	6	6.9	0.6	6	6.9	0.6
7	7.9	0.3	99	111	0.7	7	7.9	0.7	7	7.9	0.7
8	8.9	0.2	112	124	0.8	8	8.9	0.8	8	8.9	0.8
9	9.9	0.1	125	137	0.9	9	9.9	0.9	9	9.9	0.9
10	10	0	138	150	1	10	10	1	10	10	1

Table A15. Fuzzy Parameterization Scales for Social Factors

Technical Factors																				
Replicability and Flexibility (Scaled			Maturity (Scaled)			BOD Removal (%age)			COD Removal (%age)			Suspended Solids Removal (%age)			Nitrogen Removal (mg/L)			Phosphorus Removal (mg/L)		
Lower	Upper	Membership	Lower	Upper	Membership	Lower	Upper	Membership	Lower	Upper	Membership	Lower	Upper	Membership	Lower	Upper	Membership	Lower	Upper	Membership
0	0	0	0	0	0	0	0	0	0	0	0	0	0		0	0	0	0	0	0
1	1.9	0.1	1	1.9	0.1	1	10.9	0.1	1	10.9	0.1	1	10.9	0.1	1	2.9	0.1	1	2.9	0.1
2	2.9	0.2	2	2.9	0.2	11	20.8	0.2	11	20.8	0.2	11	20.8	0.2	3.9	4.8	0.2	3.9	4.8	0.2
3	3.9	0.3	3	3.9	0.3	20.9	30.7	0.3	20.9	30.7	0.3	20.9	30.7	0.3	5.8	6.7	0.3	5.8	6.7	0.3
4	4.9	0.4	4	4.9	0.4	30.8	40.6	0.4	30.8	40.6	0.4	30.8	40.6	0.4	7.7	8.6	0.4	7.7	8.6	0.4
5	5.9	0.5	5	5.9	0.5	41.6	50.5	0.5	41.6	50.5	0.5	41.6	50.5	0.5	9.6	10.5	0.5	9.6	10.5	0.5
6	6.9	0.6	6	6.9	0.6	50.6	60.4	0.6	50.6	60.4	0.6	50.6	60.4	0.6	11.5	12.4	0.6	11.5	12.4	0.6
7	7.9	0.7	7	7.9	0.7	60.5	70.3	0.7	60.5	70.3	0.7	60.5	70.3	0.7	13.4	14.3	0.7	13.4	14.3	0.7
8	8.9	0.8	8	8.9	0.8	70.4	80.2	0.8	70.4	80.2	0.8	70.4	80.2	0.8	15.3	16.2	0.8	15.3	16.2	0.8
9	9.9	0.9	9	9.9	0.9	80.3	90.1	0.9	80.3	90.1	0.9	80.3	90.1	0.9	17.2	18.1	0.9	17.2	18.1	0.9
10	10	1	10	10	1	90.2	100	1	90.2	100	1	90.2	100	1	19.1	20	1	19.1	20	1

Table A16. Fuzzy Parameterization Scales for Technical Factors

Economic Factors														
Energy Savings (MW/Year)			Operational and Maintainance cost ($$/Year)			Land Req (sq m)			Investmet Cost (Mil $$)			Sludge Disposal cost ($$/Year		
Lower	Upper	Membership	Lower	Upper	Membership	Lower	Upper	Membership	Lower	Upper	Membership	Lower	Upper	Membership
0	0	0	0	99999	1	0	0	1	0	0	1	0	0	1
35	51.5	0.1	100000	160000	0.9	5000	5500	0.9	5	7.5	0.9	35000	56500	0.9
52.5	68	0.2	160001	220000	0.8	5501	6000	0.8	8.5	10	0.8	56501	78000	0.8
69	84.5	0.3	220001	280000	0.7	6001	6500	0.7	11	12.5	0.7	78001	99500	0.7
85.5	101	0.4	280001	340000	0.6	6501	7000	0.6	13.5	15	0.6	99501	121000	0.6
102	117.5	0.5	340001	400000	0.5	7001	7500	0.5	16	17.5	0.5	121001	142500	0.5
118.5	134	0.6	400001	460000	0.4	7501	8000	0.4	18.5	20	0.4	142501	164000	0.4
135	150.5	0.7	460001	520000	0.3	8001	8500	0.3	21	22.5	0.3	164001	185500	0.3
151.5	167	0.8	520001	580000	0.2	8501	9000	0.2	23.5	25	0.2	185501	207000	0.2
168	183.5	0.9	580001	640000	0.1	9001	9500	0.1	26	27.5	0.1	207001	228500	0.1
184.5	200	1	640001	700000	0	9501	10000	0	28.5	30	0	228501	250000	0

Table A17. Fuzzy Parameterization Scales for Economic Factors

	Environmental Factors			Social Factors				Technical Factors							Economic Factors				
Alternatives	A1	A2	A3	A4	A5	A6	A7	A8	A9	A10	A11	A12	A13	A14	A15	A16	A17	A18	A19
Alt 1	0.037	0.009	0.032	0.027	0.013	0.003	0.017	0.003	0.005	0.019	0.017	0.017	0.009	0.015	0.005	0.027	0.023	0.005	0.016
Alt 2	0.021	0.004	0.012	0.025	0.012	0.015	0.009	0.005	0.001	0.016	0.016	0.016	0.010	0.009	0.002	0.025	0.005	0.015	0.017
Alt 3	0.006	0.054	0.060	0.027	0.022	0.022	0.027	0.018	0.018	0.023	0.025	0.025	0.020	0.012	0.021	0.036	0.032	0.021	0.032
Alt 4	0.032	0.009	0.037	0.024	0.017	0.010	0.010	0.002	0.006	0.020	0.018	0.018	0.012	0.014	0.016	0.028	0.002	0.011	0.016

Table A18. Fuzzy Parameterization of the Alternatives
